# Surface-cleaned black phosphorus nanosheets chelate iron and rebalance the ferroptosis-immune axis in acute kidney injury

**DOI:** 10.7150/thno.129807

**Published:** 2026-07-20

**Authors:** Wan-Lin Tan, Jin-Ming Zhu, Xiao-Xiao Xu, Jing Jia, Rong-Yu Chen, Xiong Yu, Lu-Qun Liang, Yuan-Yuan Ruan, Fang-Fang Wang, Yu-Ting Chen, Yu-Lin Peng, Jin Peng, Dan Wang, Ling-Ling Liu, Yao Ran, Bing Guo, Jian Feng, Yuan-Yuan Wang

**Affiliations:** 1 State Key Laboratory of Discovery and Utilization of Functional Components in Traditional Chinese Medicine, Guizhou Medical University; 2 Department of Pathophysiology, School of Basic Medical Sciences, Guizhou Medical University, Anshun, Guizhou 561113, China; 3 Guizhou Provincial Key Laboratory of Pathogenesis and Prevention of Common Chronic Diseases, Guizhou Medical University, No. 6 Ankang Avenue, Gui’an New District, Guizhou, 561113, China.; 4 Department of Intensive Care Unit, The Affiliated Hospital of Guizhou Medical University, No. 28, Guiyi Road, Yunyan District, Guiyang, Guizhou 550001, China; 5 Department of Obstetrics, The Affiliated Hospital of Guizhou Medical University, No. 28, Guiyi Road, Yunyan District, Guiyang, Guizhou 550001, China; 6 School of Basic Medical Sciences, Guizhou Medical University, Guiyang 561113, China

**Keywords:** Acute kidney injury, surface-cleaned black phosphorus nanosheets, ferroptosis, iron homeostasis, macrophage polarization

## Abstract

**Rationale:**

Nanomaterials have been explored for acute kidney injury (AKI) therapy because of their ability to scavenge reactive oxygen species (ROS) and reduce oxidative stress. Black phosphorus nanosheets (BPNSs) show renoprotective potential, but spontaneous surface oxidation may weaken their redox activity and therapeutic efficacy. It remains unclear whether surface engineering can improve the biological activity of BPNSs and modulate ferroptosis and immune activation during AKI.

**Methods:**

Surface-cleaned BPNSs (sc-BPNSs) were prepared by hydrogen reduction and characterized. Fluorescence-labeled sc-BPNSs were used to examine biodistribution, and therapeutic efficacy was evaluated in mice with AKI. *In vitro*, mouse renal tubular epithelial cells (mRTECs) were subjected to hypoxia/reoxygenation (H/R). Ferroptosis and iron homeostasis were assessed using biochemical, histological, and imaging assays. RNA sequencing was performed in mRTECs and macrophages to identify pathways affected by sc-BPNSs. A tubular epithelial cell–macrophage co-culture model was used to examine intercellular crosstalk after injury.

**Results:**

Hydrogen reduction produced sc-BPNSs with reduced surface oxidation, stronger antioxidant activity, and improved renal accumulation. After intravenous administration, sc-BPNSs preferentially accumulated in injured kidneys, reduced ROS accumulation and lipid peroxidation, alleviated tubular injury, and improved renal function. Transcriptomic analysis showed that sc-BPNSs affected pathways related to oxidative stress, iron metabolism, and immune regulation. Mechanistically, sc-BPNSs restored iron homeostasis by sequestering labile iron and regulating transferrin receptor 1/2 (TFR1/TFR2) and ferroportin (FPN1), thereby suppressing ferroptosis *in vitro* and *in vivo*. sc-BPNSs also inhibited Ras-MAPK signaling, reduced M1 macrophage activation, and increased M2-associated marker expression. In the co-culture model, sc-BPNSs attenuated the ferroptotic and inflammatory crosstalk between injured tubular epithelial cells and macrophages.

**Conclusions:**

sc-BPNSs protect against AKI by reducing oxidative stress, restoring iron homeostasis, suppressing ferroptosis, and modulating macrophage activation. These findings suggest that surface-cleaned BPNSs may provide a therapeutic approach for AKI by targeting both tubular ferroptosis and inflammatory amplification.

## Introduction

Acute kidney injury (AKI) remains a major clinical problem that can arise from ischemia, infection, or exposure to nephrotoxic agents. It is particularly common in critically ill patients, where it is associated with substantial morbidity, rapid clinical deterioration, and an increased risk of progression to chronic kidney disease (CKD) 1,2. At the cellular and tissue levels, AKI is driven by a network of pathological processes, including oxidative stress, disrupted iron homeostasis, tubular cell death, and inflammatory amplification. Together, these events lead to an abrupt loss of renal structure and function 3. Despite the considerable clinical burden of AKI, current management remains largely supportive, and no approved therapy directly targets the core injury pathways responsible for disease initiation and progression.

Because oxidative damage and iron-dependent ferroptotic injury are important contributors to AKI pathogenesis, small-molecule antioxidants, ferroptosis inhibitors, and iron chelators have been explored as possible therapeutic approaches. However, their clinical translation is frequently constrained by rapid systemic clearance, insufficient renal accumulation, limited retention within injured renal tissue, and the challenge of simultaneously modulating multiple interconnected injury pathways 4,5. Classical iron chelators, such as deferoxamine (DFO), further suffer from unfavorable pharmacokinetic properties, including a short circulating half-life and limited bioavailability, which may compromise their sustained activity *in vivo* 6,7.

Black phosphorus nanosheets (BPNSs) are a two-dimensional nanomaterial with favorable biocompatibility and inherent reactive oxygen species (ROS)-scavenging activity 8,9. Recent advances in AKI nanomedicine have shown that engineered nanosystems can modulate oxidative stress, inflammatory cell death, and renal injury 10,11. For BPNSs, their antioxidant activity is generally attributed to reactions between ROS and exposed phosphorus atoms on the nanosheet surface. This activity, however, is highly dependent on the surface chemical state of BPNSs and may be compromised by spontaneous surface oxidation. During routine preparation and processing, BPNSs are readily oxidized, which can markedly weaken their redox reactivity and metal coordination capacity 12,13.

To overcome this limitation, a post-treatment hydrogen reduction strategy was applied to convert conventionally processed, partially surface-oxidized BPNSs into surface-cleaned BPNSs (sc-BPNSs) (Figure S1A). By removing surface oxygen species, this strategy increases the proportion of exposed phosphorus atoms, thereby conferring sc-BPNSs with enhanced antioxidant and metal ion-coordinating properties and more fully releasing the structure-determined intrinsic functional advantages of BPNSs.

Among the pathogenic mechanisms of AKI, ferroptosis and macrophage-mediated inflammation have emerged as closely linked processes. Ferroptosis directly contributes to tubular epithelial injury through iron-dependent lipid peroxidation, whereas proinflammatory macrophages further amplify tubular damage and sustain the inflammatory microenvironment 14-18. Increasing evidence suggests that iron dysregulation and immune activation converge into an iron-immune axis, which may represent a therapeutically actionable vulnerability in AKI 19,20. Consistently, emerging nanotherapeutic studies have shown that AKI can be attenuated by directly scavenging ROS and/or removing pathological labile iron species in the kidney 21,22.

Preliminary studies showed that sc-BPNSs alleviated tubular injury and improved renal function in multiple AKI models, including ischemia/reperfusion, cisplatin-induced, and folic acid-induced injury. Transcriptomic analyses further suggested that sc-BPNSs affect pathways related to iron handling, oxidative stress, lipid metabolism, and inflammatory activation. Based on these observations, sc-BPNSs were hypothesized to protect against AKI through dual actions in the tubular and immune compartments. Specifically, sc-BPNSs may restore iron homeostasis and suppress ferroptotic injury in renal tubular epithelial cells while attenuating proinflammatory macrophage activation. To test this hypothesis, the effects of sc-BPNSs were examined in *in vivo* AKI models, tubular epithelial and macrophage injury systems, and epithelial cell-macrophage co-culture models.

## Materials and Methods

### Preparation of sc-BPNSs

The sc-BPNSs were prepared by electrochemical exfoliation, liquid-phase sonication, and subsequent hydrogen reduction. High-purity bulk black phosphorus served as the anode, and a graphite rod was used as the cathode. Electrochemical exfoliation was carried out in 0.01 M KOH under a constant voltage. The exfoliated material was then recovered by centrifugation and washed to remove residual electrolyte. The collected product was dispersed in N-methyl-2-pyrrolidone (NMP) and probe-sonicated in an ice-water bath to promote further layer separation. The obtained BPNSs were then annealed under a hydrogen atmosphere (200 °C、12 hours) to remove surface oxygen-containing species (Figure S1A). After reduction, the products were washed with absolute ethanol, followed by lyophilized, and stored for further using.

### Preparation of Fluorescently Labeled sc-BPNSs

Fluorescently labeled sc-BPNSs were prepared with minor modifications to a previously reported method. Ultrapure water was first boiled for approximately 10 min and then purged with argon while cooling to room temperature to reduce dissolved oxygen. The total deoxygenation procedure lasted approximately 2 h. sc-BPNSs (5 mg) were dispersed in 50 mL of deoxygenated ultrapure water with gentle sonication. Cy5.5-PEG fluorescent dye (Xi’an Ruixi Biological Technology Co., Ltd., China) was added at a Cy5.5-PEG:sc-BPNSs mass ratio of 1:2, corresponding to 2.5 mg dye for 5 mg sc-BPNSs. The suspension was stirred at room temperature for 24 h to allow dye adsorption onto the sc-BPNSs. The suspension was then centrifuged at 10,000 rpm for 10 min. The precipitate was washed three times with distilled water to remove unbound dye. The labeled product was freeze-dried and re-dispersed in deoxygenated ultrapure water at 1 mg/mL before use. The binding mode between sc-BPNSs and the fluorescent dyes, including Cy5.5-PEG, FITC, and ICG-PEG, was not examined in detail in the present study. Based on previous reports, the labeling process was considered to mainly depend on non-covalent interactions.

### Characterization of sc-BPNSs

The morphology of sc-BPNSs was observed by transmission electron microscopy (TEM; JEM-2100, JEOL, Japan). Nanosheet thickness was measured using atomic force microscopy (AFM; Dimension Icon, Bruker, USA). X-ray diffraction (XRD; SmartLab, Rigaku, Japan) was performed to examine the crystalline structure, and X-ray photoelectron spectroscopy (XPS; K-Alpha, Thermo Scientific, USA) was used to analyze surface chemical composition. Hydrodynamic size and zeta potential were determined by dynamic light scattering (DLS; Zetasizer Nano ZS, Malvern, UK).

### Antioxidant Activity Assays of sc-BPNSs

The antioxidant activity of sc-BPNSs was tested by measuring their scavenging capacity against superoxide anion (O₂⁻), hydroxyl radical (•OH), 2,2-diphenyl-1-picrylhydrazyl (DPPH), and 2,2′-azino-bis(3-ethylbenzothiazoline-6-sulfonic acid) (ABTS) radicals. Commercial assay kits were used according to the manufacturers’ protocols. BPNSs and sc-BPNSs were dispersed at 0, 5, 10, 20, and 50 μg/mL and added to the corresponding radical-generating reaction systems. After the reactions were completed, absorbance was recorded with a UV-Vis spectrophotometer, and radical scavenging rates were calculated. Untreated samples served as blank controls. The antioxidant performance of sc-BPNSs was compared with that of conventional oxidized BPNSs.

### Animal Model of Acute Kidney Injury and sc-BPNSs Treatment

Male C57BL/6J mice aged 6-8 weeks and weighing 20 ± 2 g were purchased from Huafukang Biotechnology Co., Ltd. (Beijing, China; license No. SCXK[Beijing]2019-0008). Mice were housed under specific pathogen-free conditions with a 12 h light/dark cycle at 25 ℃ and 40%-60% humidity, with free access to food and water. After 7 days of acclimatization, mice were anesthetized by intraperitoneal injection of 1% pentobarbital sodium at 0.01 mL/g body weight before all procedures. All animal experiments were approved by the Institutional Animal Care and Use Committee of Guizhou Medical University (approval No. 2200402).

For the bilateral renal ischemia/reperfusion (I/R) model, both renal pedicles were bluntly separated and clamped for 30 min using atraumatic microvascular clamps under temperature-controlled conditions at 37.5 °C, followed by clamp release to allow reperfusion. Sham-operated mice underwent the same surgical procedure without vascular occlusion. In the I/R + sc-BPNS group, sc-BPNSs were administered via tail vein injection at 3 mg/kg in 120 μL phosphate-buffered saline (PBS) 30 min before ischemia. Mice in the sham and I/R groups received an equal volume of PBS.

For the unilateral renal I/R model used for *in vivo* biodistribution analysis, the left renal pedicle was isolated and clamped for 30 min under temperature-controlled conditions at 37.5 °C, followed by reperfusion after clamp release. Sham-operated mice underwent the same surgical exposure without clamping. For fluorescence imaging experiments, labeled sc-BPNSs were administered via tail vein injection before ischemia as described above.

For the folic acid (FA; 200 mg/kg; MedChemExpress, HY-16637, USA) and cisplatin (Cis; 20 mg/kg; APExBIO, A8321, USA)-induced AKI models, 8-week-old C57BL/6J mice were acclimated for 1 week and randomly assigned to six groups: sham for the FA experiment, FA, FA + sc-BPNSs, sham for the Cis experiment, Cis, and Cis + sc-BPNSs. The FA experiment included six mice per group, and the Cis experiment included five mice per group. Mice in the sham groups received equal volumes of normal saline by tail vein and intraperitoneal injection. FA, Cis, and sc-BPNSs were dissolved in saline before use. In the FA + sc-BPNS and Cis + sc-BPNS groups, sc-BPNSs were administered by tail vein injection at 3 mg/kg 30 min before intraperitoneal injection of FA or Cis. Kidneys and blood samples were collected 48 h after FA injection and 72 h after Cis injection.

### Cell Culture and Injury Modeling in Renal Tubular Epithelial Cells

Mouse renal tubular epithelial cells (mRTECs; Huatuo Biotechnology Co., Ltd.) were cultured in low-glucose DMEM containing 1 g/L glucose and supplemented with 10% fetal bovine serum (FBS) and 1% penicillin-streptomycin at 37 ºC in a humidified incubator with 5% CO₂. Hypoxia/reoxygenation (H/R) modeling was initiated when cells reached approximately 60% confluence. For H/R induction, cells were cultured in serum-free DMEM under 1% O₂, 5% CO₂, and 94% N₂ for 4 h, followed by reoxygenation in complete medium for 24 h under normoxic conditions. The experimental groups included (1) normal control (NC), (2) H/R injury, (3) H/R + sc-BPNSs at 20 μg/mL, (4) H/R + deferoxamine (DFO; 100 μM; APExBIO, B6068, USA), and (5) H/R + N-acetyl-L-cysteine (NAC; 2 mM; MedChemExpress, HY-B0215, USA). In the sc-BPNSs, DFO, and NAC groups, treatments were administered 1 h before hypoxia.

Erastin-induced ferroptosis was modeled by treating cells with 5 nM erastin (Selleck, S7242, USA) for 24 h. The experimental groups were as follows: (1) NC, (2) erastin, (3) erastin + sc-BPNSs at 20 μg/mL, and (4) erastin + NAC at 2 mM, with sc-BPNSs added simultaneously with erastin. Fe²⁺/Fe³⁺ overload models were established in 70%-80% confluent mRTECs cultured in low-glucose DMEM containing 2% FBS. The experimental groups were as follows: (1) NC, (2) Fe²⁺ treatment with 50 μM FeCl₂, (3) Fe²⁺ + sc-BPNSs, (4) Fe³⁺ treatment with 150 μM FeCl₃, and (5) Fe³⁺ + sc-BPNSs. All treatments were conducted for 24 h.

For the transfection + H/R model, plasmid transfection was performed using Lipofectamine 3000 (Invitrogen, L3000008) 6 h before reoxygenation, followed by H/R treatment as described above. The experimental groups were as follows: (1) transfection + H/R, (2) transfection + H/R + sc-BPNSs, and (3) empty vector control.

### Culture and Stimulation of RAW264.7 Macrophages

RAW264.7 cells, a mouse monocyte/macrophage leukemia cell line, were obtained from Procell (China) and cultured in high-glucose DMEM containing 4.5 g/L glucose, supplemented with 10% FBS and 1% penicillin/streptomycin. Cells were maintained at 37 ºC in a humidified incubator with 5% CO₂. The experimental groups were as follows: (1) NC, (2) H/R, (3) lipopolysaccharide (LPS), (4) interleukin-4 (IL-4), (5) H/R + sc-BPNSs, (6) LPS + sc-BPNSs, and (7) IL-4 + sc-BPNSs.

For the H/R model, RAW264.7 cells were exposed to hypoxia for 4 h, followed by reoxygenation for 24 h. For inflammatory stimulation, cells were treated with LPS (100 ng/mL; Proteintech, 14011S) for 24 h. For alternative activation, cells were treated with recombinant human IL-4 protein (20 ng/mL; Proteintech, HZ-1004) for 24 h. In the corresponding intervention groups, sc-BPNSs at 20 μg/mL were added before model induction. Cells were then collected for subsequent Western blotting, flow cytometric, and molecular analyses.

### Isolation and Differentiation of Bone Marrow-Derived Macrophages

Bone marrow-derived macrophages (BMDMs) were isolated from male C57BL/6J mice aged 6–8 weeks. After euthanasia, femurs and tibias were collected under sterile conditions, and bone marrow was flushed with sterile PBS. The cell suspension was filtered, centrifuged, treated with red blood cell lysis buffer, washed, and resuspended in α-MEM containing 10% heat-inactivated FBS and 1% penicillin/streptomycin. Cells were plated overnight to remove rapidly adherent cells, and non-adherent cells were collected and cultured in non-tissue-culture-treated dishes with recombinant mouse M-CSF (50 ng/mL; NovoProtein, CB34, China). Fresh M-CSF-containing medium was replaced every other day. After 5-7 days, mature adherent macrophages with typical morphology were obtained and, where indicated, verified by F4/80 and CD11b expression. The resulting M0 macrophages were washed, resuspended, and replated at 5 × 10⁵ cells for subsequent experiments.

The experimental groups were as follows: (1) NC, (2) H/R, (3) LPS, (4) IL-4, (5) H/R + sc-BPNSs, (6) LPS + sc-BPNSs, (7) IL-4 + sc-BPNSs, (8) H/R + RAS inhibitor Abd-7 (20 μM; MedChemExpress, HY-122862), (9) LPS + Abd-7, and (10) IL-4 + Abd-7.

For the H/R model, BMDMs were exposed to hypoxia for 4 h, followed by reoxygenation for 24 h. For inflammatory stimulation, cells were treated with LPS (100 ng/mL; Proteintech, 14011S) for 24 h. For alternative activation, cells were treated with recombinant human IL-4 protein (20 ng/mL; Proteintech, HZ-1004) for 24 h. In the corresponding intervention groups, sc-BPNSs at 20 μg/mL or Abd-7 were added before model induction. After treatment, cells were collected and processed for flow cytometric analysis.

### *In vivo* Biodistribution of sc-BPNSs

For tissue distribution analysis, sc-BPNSs were labeled with Cy5.5-PEG to obtain Cy5.5-sc-BPNSs. The labeled nanosheets were injected into C57BL/6 mice through the tail vein at 3 mg/kg 30 min before renal I/R surgery, with three mice assigned to each group. Mice were sacrificed at 1, 6, 24, and 48 h after surgery. The heart, liver, spleen, lung, and kidneys were then harvested for *ex vivo* fluorescence imaging. Cy5.5 signals were recorded using a small animal imaging system (NEWTON FT 500, Vilber, USA). The same imaging settings were used for all samples, and fluorescence intensity was measured with Kuant software to compare organ distribution and renal accumulation.

In the unilateral renal I/R model, the left renal pedicle was clamped for 30 min and then released to allow reperfusion. Cy5.5-sc-BPNSs were injected via the tail vein at 3 mg/kg 30 min before surgery. At 1, 6, and 24 h after surgery, mice were sacrificed, and both kidneys were collected for *ex vivo* fluorescence imaging and quantitative analysis.

For whole-body surface imaging, sc-BPNSs were labeled with indocyanine green (ICG-sc-BPNSs) and injected via the tail vein at 3 mg/kg 30 min before unilateral left renal I/R surgery or sham operation. At 1, 6, and 24 h after surgery, fluorescence signals were acquired *in vivo* from both dorsal and ventral views using the same imaging system. Because renal enrichment could not be clearly resolved by whole-body surface imaging alone, kidneys were further harvested in the corresponding Cy5.5-sc-BPNSs experiments for *ex vivo* fluorescence analysis.

### Cellular Uptake of sc-BPNSs in mRTECs

To visualize intracellular uptake, Cy5.5-sc-BPNSs at 20 μg/mL were added to mRTECs cultured on glass-bottom dishes. After cell attachment, the medium was changed to serum-free medium containing Cy5.5-sc-BPNSs and Hoechst 33342 diluted at 1:4000 (CA1120, Solarbio, China). Following a 30-min incubation at 37 ℃, the staining medium was replaced with fresh medium, and cells were imaged with a laser confocal microscope (Olympus, Japan) to determine the intracellular distribution of Cy5.5-sc-BPNSs.

### *In vivo* Subacute Toxicity Evaluation of sc-BPNSs

To evaluate subacute toxicity, C57BL/6 mice were randomly divided into control and treatment groups, with at least six mice included per group. Mice in the treatment group received tail vein injections of sc-BPNSs at 3 mg/kg every 3 days for 28 days. At the end of the study, mice were euthanized, and major organs, including heart, liver, spleen, lung, and kidney, were collected, fixed, embedded, sectioned, and stained with hematoxylin and eosin (H&E) for histopathological examination. Blood samples were collected for serum biochemical analysis using an automatic biochemical analyzer (AU5800, Beckman Coulter, Roche). Serum creatinine (sCr), alanine aminotransferase (ALT), aspartate aminotransferase (AST), and creatine kinase-MB (CK-MB) were quantified to assess renal, hepatic, and cardiac toxicity.

### Renal Function Assessment

At 24 h after surgery, glomerular filtration rate (GFR) was measured by tail vein injection of FITC-sinistrin followed by real-time monitoring of transdermal fluorescence decay using the MediBeacon TGFR system (MediBeacon GmbH, Germany). Additionally, blood was collected from the abdominal aorta, and sCr and blood urea nitrogen (BUN) levels were analyzed using an automatic biochemical analyzer (AU5800, Beckman Coulter).

### Hematoxylin and Eosin Staining

Kidneys were harvested at 24 h after surgery, fixed in 4% paraformaldehyde, embedded in paraffin, and sectioned at a thickness of 4 μm. After deparaffinization and rehydration, sections were stained using a hematoxylin and eosin (H&E) kit (Solarbio, G1120, China), followed by bluing under running water, graded ethanol dehydration, xylene clearing, and mounting. Renal tubular dilation, epithelial necrosis, brush border loss, and inflammatory infiltration were examined under a light microscope (Olympus BX53, Japan). For semiquantitative analysis, ten random fields from the corticomedullary junction were evaluated at 200× magnification by two blinded pathologists using the following scoring criteria: 0, normal; 1, injury < 25%; 2, injury 25-50%; 3, injury 50-75%; 4, injury > 75%.

### Periodic Acid-Schiff Staining

After fixation and sectioning as described above, tissue sections underwent peroxidase blocking and rehydration. Sections were stained using a periodic acid-Schiff (PAS) kit (Solarbio, G1281, China), followed by hematoxylin counterstaining, bluing, dehydration, clearing, and mounting. Sections were examined under light microscopy to assess brush borders and basement membrane integrity.

### Western Blot Analysis

Total protein was extracted using radioimmunoprecipitation assay (RIPA) buffer and quantified using a bicinchoninic acid (BCA) protein assay kit (Beyotime, P0010, China). Equal amounts of protein, ranging from 30 to 50 μg, were separated by SDS-PAGE and transferred onto PVDF membranes. After blocking at room temperature for 1 h, membranes were incubated overnight at 4 ºC with primary antibodies, all diluted at 1:1000 unless otherwise stated. The primary antibodies included Ngal (Abclonal, A2092, China), KIM-1 (Bioss, bs-2713R, China), MCP-1 (ZenBio, 220691, China), glutathione peroxidase 4 (GPX4; ZenBio, 381958, China), FTH1 (ZenBio, 381204, China), TFR2 (Boster, MA02353, China), TFR1 (ZenBio, 381603, China), FPN1 (Proteintech, 26601-1-AP, China), IRP1 (Proteintech, 12406-1-AP, China), iNOS (Bioss, bs-0162R, China), CD206 (Proteintech, 18704-1-AP, China), TNF-α (Cell Signaling Technology, #11948, USA), phospho-P38 (Thr180/Tyr182; Proteintech, 28796-1-AP, China), P38 (Proteintech, 66234-1-Ig, China), kRas (Proteintech, 12063-1-AP, China), phospho-RAF (Ser338/Thr341; Cell Signaling Technology, #9427, USA), phospho-MEK (Ser217/221; Cell Signaling Technology, #3958, USA), MEK (Proteintech, 11049-1-AP, China), phospho-ERK (Thr202/Tyr204; Proteintech, 28733-1-AP, China), ERK (Proteintech, 83533-1-RR, China), STAT3 (ZenBio, R22785, China), phospho-STAT3 (Ser727; ZenBio, 310019, China), and β-actin (1:5000, Biopm, PMK058M/S, China), which served as the internal reference. The following day, HRP*-*conjugated secondary antibodies, including goat anti-rabbit IgG (H+L), HRP (Biopm, PMK-014-090M, China) and goat anti-mouse IgG (H+L)-HRP (Biopm, PMK-014-091M, China), were applied at 1:5000 for 1 h at room temperature. Bands were visualized using ECL reagent and quantified using ImageJ, with β-actin used as the loading control.

### TUNEL Staining

Cell apoptosis in kidney tissue was evaluated using a terminal deoxynucleotidyl transferase dUTP nick-end labeling (TUNEL) Apoptosis Assay Kit (Promega, G3250, USA). After deparaffinization, sections were incubated with 20 μg/mL proteinase K for 15 min at room temperature, followed by the TUNEL reaction according to the manufacturer’s instructions. After counterstaining with 4’,6-diamidino-2-phenylindole (DAPI), the sections were mounted and examined using a laser confocal microscope (Olympus, Japan). For each section, six randomly selected fields in the corticomedullary junction were analyzed, and the proportion of TUNEL-positive cells was calculated.

### Immunohistochemistry

Paraffin-embedded kidney sections were deparaffinized, rehydrated, and treated with 3% hydrogen peroxide to quench endogenous peroxidase activity. Antigen retrieval was carried out by microwave heating in citrate buffer. Once the sections had cooled to room temperature, they were blocked with 10% donkey serum for 1 h and incubated overnight at 4 ºC with primary antibodies against F4/80 (1:200), GPX4 (1:200), or 4-hydroxynonenal (4-HNE, 1:200; Bioss, bs-6313R, China). Following washes with Tris-buffered saline containing 0.1% Tween-20 (TBST), the sections were incubated with polymer horseradish peroxidase (HRP)-conjugated secondary antibodies. Color development was performed with 3,3′-diaminobenzidine (DAB; Gene Tech, GK500705, China). The sections were then counterstained with hematoxylin, dehydrated, cleared, mounted, and photographed under a light microscope (Olympus, Japan).

### FerroOrange Fluorescent Probe Staining for Fe²⁺

Intracellular ferrous iron (Fe²⁺) was detected using FerroOrange staining. mRTECs were seeded in glass-bottom dishes and cultured overnight before model induction. H/R injury was induced by 4 h of hypoxia followed by 24 h of reoxygenation, whereas the ferroptosis model was established by treating cells with 5 nM erastin for 24 h. After three PBS washes, cells were incubated with FerroOrange (1 μM; Dojindo, F374) diluted in serum-free DMEM for 30 min at 37 ºC. After staining, cells were observed using a laser confocal microscope (Olympus, Japan) for Fe²⁺ signal detection.

### Liperfluo Fluorescent Probe Staining for Lipid Peroxidation

Lipid ROS was assessed using Liperfluo staining (Dojindo, L248). mRTECs were seeded in glass-bottom dishes, cultured overnight, and subjected to H/R or erastin treatment as described above. After treatment, cells were incubated with Liperfluo working solution at 3 μL/mL for 30 min at 37 ºC, washed twice, and imaged using confocal microscopy. Green fluorescence indicated lipid peroxidation.

### Cell Viability Assay

Cell viability was assessed using the Cell Counting Kit-8 (CCK-8) assay. mRTECs were seeded in 96-well plates at 1 × 10⁴ cells/well and cultured for 24 h. Cells were then treated with sc-BPNSs at 0-80 μg/mL, Fe²⁺, or Fe³⁺ at 0-400 μM for 24 h. Subsequently, 10 μL of CCK-8 reagent (ApexBio, S1010, USA) was added to each well and incubated for 2 h. Absorbance was measured at 450 nm using a microplate reader. All conditions were tested in triplicate, and results were expressed as the mean ± SD.

### Flow Cytometry Analysis

#### Detection of Macrophage M1/M2 Phenotypes

RAW264.7 cells were subjected to H/R, consisting of 4 h hypoxia followed by 24 h reoxygenation, or treated with LPS at 100 ng/mL for 24 h. After two washes with PBS, cells were incubated with Fc receptor blocker for 10 min. Subsequently, cells were stained with PerCP*-*conjugated anti-F4/80 antibody (BioLegend, 123125, USA), FITC-conjugated anti-I-A/I-E antibody as an M1 marker (BioLegend, 107605, USA), and PE-conjugated anti-CD206 antibody as an M2 marker (BioLegend, 141707, USA) for 30 min in the dark. After two PBS washes, samples were analyzed using a BD FACSCanto II cytometer, with 10,000 events recorded per sample. Data were analyzed using FlowJo software.

#### Intracellular ROS Detection

After treatment, mRTECs were collected, washed with PBS, and incubated with 10 μM DCFH-DA (Beyotime, S0033, China) in serum-free medium for 30 min at 37 ºC in the dark, with gentle mixing every 10 min. Cells were then washed twice with PBS and immediately analyzed using a BD FACSCanto II cytometer. For each sample, 10,000 events were acquired, and fluorescence intensity was analyzed with NovoExpress software.

#### Preparation of Kidney Single-Cell Suspensions

Kidneys were collected from mice and rinsed with pre-chilled calcium- and magnesium-free PBS (Thermo Fisher Scientific, 10010049, USA). The renal capsule, surrounding fat, and pelvis were carefully removed. The remaining tissue was cut into approximately 1 mm³ pieces and transferred into 5 mL of pre-warmed digestion buffer containing collagenase IV (Thermo Fisher Scientific, 17104019, USA) and DNase I (Servicebio, G3342, China). Digestion was performed at 37 ºC for 30-40 min, with gentle mixing every 5 min. The digestion reaction was stopped by adding an equal volume of staining buffer, consisting of PBS supplemented with 2% FBS. The cell suspension was passed through a 70 μm strainer (Corning, CLS431751, USA), centrifuged at 300 × g for 5 min at 4 ºC, and treated with red blood cell lysis buffer (Servicebio, G2015, China) for 3-5 min. After a second filtration through a 40 μm strainer, cells were resuspended in staining buffer at approximately 1 × 10⁶ cells/mL. All steps were carried out on ice or at 4 ºC, except for enzymatic digestion.

#### *In vivo* Detection of FITC-sc-BPNS Uptake in Renal Cell Subsets

To evaluate the cellular uptake of sc-BPNSs *in vivo*, mice were intravenously administered with FITC-labeled sc-BPNSs (FITC-sc-BPNSs). After 24 h, kidneys were harvested and enzymatically dissociated into single-cell suspensions. Cells were kept on ice and incubated with Fc receptor blocker for 10 min before antibody staining. The staining panel included Pacific Blue anti-mouse CD45 antibody (BioLegend, 157212, USA; 1:100), APC anti-mouse CD326 (EpCAM) antibody (BioLegend, 118213, USA; 1:100), PerCP-conjugated anti-mouse F4/80 antibody (BioLegend, 123125, USA; 1:100), and PE anti-mouse CD192 (CCR2) antibody (BioLegend, 150609, USA; 1:100). Staining was performed for 30 min in the dark.

After two washes, samples were run on a BD FACSVerse flow cytometer (BD Biosciences, USA), and the data were analyzed using FlowJo software. Tubular epithelial cells were identified as CD45⁻EpCAM⁺ cells. Infiltrating macrophages were gated as CD45⁺F4/80⁺CCR2⁺ cells, whereas resident macrophages were gated as CD45⁺F4/80^high^CCR2⁻ cells. The percentage of FITC-positive cells was then calculated within each gated population.

#### Flow Cytometric Analysis of Infiltrating and Resident Macrophage Polarization in Kidney Tissues

Macrophage polarization in kidney tissue was analyzed using a separate aliquot of kidney single-cell suspensions. After Fc receptor blocking, cells were stained with PerCP-conjugated anti-mouse F4/80 antibody (BioLegend, 123125, USA; 1:100), PE anti-mouse CD192 (CCR2) antibody (BioLegend, 150609, USA; 1:100), FITC-conjugated anti-I-A/I-E antibody (BioLegend, 107605, USA; 1:100), and APC-conjugated anti-CD206 antibody (BioLegend, 141707, USA; 1:100). Cells were incubated with antibodies for 30 min in the dark and washed twice before flow cytometric acquisition on a BD FACSVerse cytometer. FlowJo software was used for gating and data analysis. Infiltrating macrophages were defined as F4/80⁺CCR2⁺ cells, and resident macrophages were defined as F4/80^high^CCR2⁻ cells. Within each macrophage subset, M1-like cells were defined as MHC II⁺CD206⁻, whereas M2-like cells were defined as MHC II⁻CD206⁺.

### Measurement of GSSG/GSH Ratio

The GSSG/GSH ratio was measured in kidney tissues and mRTECs using a commercial GSH/GSSG assay kit (Dojindo, G263, Japan). Left kidney samples were collected 24 h after surgery and homogenized in PBS. Treated cells were collected in parallel. Tissue homogenates and cell lysates were mixed with 5% 5-sulfosalicylic acid to remove proteins. After centrifugation, the supernatants were used for the measurement of total glutathione (tGSH) and GSSG following the manufacturer’s protocol. GSH was calculated as GSH = tGSH - 2 × GSSG, and the GSSG/GSH ratio was used to reflect the redox state. Absorbance was recorded at 412 nm and normalized to protein content.

### Malondialdehyde Assay

MDA levels in kidney tissues were measured with a fluorescence-based malondialdehyde assay kit (Dojindo, M496, Japan). Left kidney tissue was collected 24 h after surgery. For each sample, 10 mg of tissue was homogenized on ice in 300 μL of antioxidant PBS solution and centrifuged at 10,000 × g for 5 min. Then, 100 μL of the supernatant was mixed with 100 μL of lysis buffer and incubated at room temperature for 5 min. Next, 250 μL of working solution was added, and the mixture was heated at 95 ºC for 15 min. After cooling on ice, samples were centrifuged again at 10,000 × g for 10 min. A 200 μL aliquot of the supernatant was transferred to a 96-well plate, and fluorescence was measured at 540 nm excitation and 590 nm emission. MDA concentrations were determined from a standard curve and normalized to tissue weight.

### Quantitative Real-Time PCR

Total RNA was isolated with TRIzol reagent (Invitrogen, 15596018). RNA concentration and purity were checked using a NanoDrop 2000 spectrophotometer (Thermo Scientific). cDNA was synthesized with the PrimeScript RT kit (Takara, RR037A). Quantitative real-time PCR (qPCR) was performed on a QuantStudio 3 system (ABI) using TB Green® Premix Ex Taq™ II (Takara, RR820A). Each sample was run in triplicate, and melting curve analysis was included to verify amplification specificity. Relative gene expression was calculated using the 2^-ΔΔCt^ method, with GAPDH as the internal control.

Primer sequences used for qPCR were synthesized by Sangon Biotech and are listed below Table 1.

### Plasmid Transfection

Overexpression plasmid transfection was performed in mRTECs using Lipofectamine™ 3000 (Invitrogen, L3000015). When cells reached 60-70% confluence, plasmid DNA and transfection reagents were diluted in Opti-MEM (Gibco, 31985070) and complexed according to the manufacturer’s protocol. The transfection complexes were added to cells and incubated at 37 ℃ for 4-6 h. The medium was then replaced with complete DMEM, and cells were cultured for another 24 h before subsequent H/R treatment or sample collection for RNA and protein analyses. The *Tfr2* overexpression plasmid was constructed and verified by GeneChem (Shanghai, China).

### siRNA Transfection

Murine mRTECs were transfected with small interfering RNAs (siRNAs) targeting *Irp1* or *Stat3*, or with the corresponding negative control siRNA, at a final concentration of 50 nM using Lipofectamine™ 3000 according to the manufacturer’s instructions. Briefly, when cells reached 60%-70% confluence, siRNAs and transfection reagent were diluted in Opti-MEM, gently mixed, and incubated to allow complex formation before being added to cells. After incubation at 37 ºC for 4-6 h, the medium was replaced with complete DMEM, and cells were cultured for an additional 24 h before subsequent H/R treatment or collection for RNA and protein analyses. The siRNAs were purchased from Shanghai Yile Biotechnology Co., Ltd. (Shanghai, China).

### RNA Immunoprecipitation Assay

RNA immunoprecipitation (RIP) assays were performed using the Dynabeads™ Protein A Immunoprecipitation Kit (Invitrogen, 10006D) according to the manufacturer’s protocol. Treated mRTECs were lysed with RIP lysis buffer, and the clarified lysates were incubated overnight at 4 ºC with Dynabeads coupled to anti-IRP1 antibody (Proteintech, 12406-1-AP). Dynabeads coupled to normal rabbit IgG were used as the negative control. After incubation, the beads were washed to reduce nonspecific binding, followed by proteinase K digestion and RNA purification. The recovered RNA was reverse-transcribed into cDNA, and qPCR was used to determine the enrichment of *Tfr1*, *Fth1*, and *Fpn1* mRNAs. Enrichment in the anti-IRP1 pulldown was calculated by the ΔCt method, with the IgG pulldown serving as the control.

### Aconitase 1 Activity Assay

ACO1 activity in mRTECs was determined with a commercial aconitase activity assay kit (Boxbio, AKAC008M). The experimental groups were as follows: (1) NC, (2) H/R, (3) H/R + sc-BPNSs, and (4) H/R + DFO. For each group, 5 × 10⁶ cells were kept on ice and homogenized in 1 mL of Reagent 1 supplemented with 10 μL of Reagent 3. The homogenates were sonicated at 300 W for 15 cycles, with each cycle consisting of 3 s of sonication followed by a 9 s interval. After sonication, samples were centrifuged at 11,000 × g for 15 min at 4 ºC, and the supernatants were collected for enzymatic measurement. For each reaction, 180 μL of Reagent 4 and 20 μL of sample were added to a 96-well UV plate. Absorbance at 240 nm was measured at 10 s (A₁) and 310 s (A₂) at 25 ºC. The absorbance change was calculated as ΔA = A₂ - A₁ and used to determine ACO1 activity according to the kit protocol. Enzyme activity was normalized to total cell number. Samples were handled at low temperature throughout the procedure to minimize loss of enzymatic activity.

### Prussian Blue Staining

Kidney tissues collected 24 h after surgery were processed for Prussian blue staining to examine iron deposition. Samples were fixed in 4% paraformaldehyde, embedded in paraffin, and cut into 4 μm sections. After deparaffinization and rehydration, the sections were incubated for 30 min at room temperature in a freshly prepared 1:1 mixture of 2% potassium ferrocyanide and 2% hydrochloric acid. The slides were rinsed with distilled water, counterstained with eosin for 4 h, dehydrated through graded alcohols, cleared in xylene, and coverslipped. Iron deposits were examined under a light microscope.

### *In vitro* Validation of Fe³⁺ Chelation by sc-BPNSs

Ferric chloride hexahydrate (FeCl₃·6H₂O; 16.6 mg) was dissolved in 10 mL of ultrapure water to obtain a Fe³⁺ stock solution. In parallel, 1 mg of sc-BPNSs was dispersed in 1 mL of ultrapure water using ultrasonication to form a homogeneous suspension at 1 mg/mL. The UV-Vis absorption spectrum of Fe³⁺ solution was recorded before adding sc-BPNSs. The sc-BPNS suspension was then added to the Fe³⁺ solution stepwise in 5 μL aliquots. Each addition was separated by a 1 min interval with brief vortexing, and the spectrum was collected after each step. After titration, the sc-BPNSs-Fe³⁺ mixture was centrifuged at 20,000 rpm for 30 min. The pellet was washed three times with acetone, vacuum-dried, and subjected to XPS analysis.

For the DPPH assay, the Fe³⁺/sc-BPNSs complex obtained after 120 min of incubation was used for radical scavenging analysis. The absorption spectrum at 120 min was compared with the initial spectrum recorded at 0 min.

### Transmission Electron Microscopy for Mitochondria

Kidney tissue samples were fixed for 1 h in 0.1 M phosphate buffer containing 1% osmium tetroxide and 1% potassium ferrocyanide, and then stained with 1% uranyl acetate for contrast enhancement. Samples were dehydrated through a graded ethanol series and embedded in Epon resin. After polymerization at 65 ℃ for 48 h, ultrathin sections with a thickness of approximately 7 nm were cut using a Leica ultramicrotome, mounted on copper grids, counterstained with lead citrate, and examined by transmission electron microscopy (TEM). Images of mitochondrial ultrastructure were acquired using an Eagle 4k CCD camera.

### RNA Sequencing

After the indicated treatments, total RNA was isolated from mRTECs and RAW264.7 cells for transcriptomic analysis. Each group contained three biological replicates. RNA sequencing (RNA-seq) for mRTECs was completed by GENE DENOVO (Guangzhou, China), whereas RNA-seq for RAW264.7 cells was completed by Tianhao Biotechnology (Shanghai, China). Cells were washed once with ice-cold PBS, and 1 mL of TRIzol reagent was added for every 10 cm² of culture area. The lysates were pipetted repeatedly, transferred to RNase-free cryotubes, snap-frozen in liquid nitrogen, and stored at -80 ºC. Samples were shipped on dry ice and arrived at the sequencing facilities within 48 h. Library construction, sequencing, differential expression analysis, and bioinformatics analysis were carried out by the corresponding service providers.

### Prediction of STAT3 Binding Sites Using JASPAR

Potential STAT3 binding sites in the mouse *Tfr2* promoter were screened using the JASPAR database. The sequence from 2000 bp upstream to 200 bp downstream of the *Tfr2* transcription start site (TSS) was entered for motif prediction. Candidate Stat3-binding motifs were ranked according to their prediction scores, and the top five sites were selected for subsequent analysis and schematic presentation.

### Co-culture of mRTECs and RAW264.7 Cells

An indirect Transwell-based co-culture system was used to examine the interaction between mRTECs and RAW264.7 macrophages without direct cell-to-cell contact. mRTECs were seeded in 6-well plates at 1 × 10^5^ cells/well in low-glucose DMEM containing 10% FBS and 1% penicillin/streptomycin, whereas RAW264.7 cells were seeded in co-culture inserts at 1 × 10^5^ cells/well in high-glucose DMEM supplemented with 10% FBS and 1% penicillin/streptomycin. After overnight culture, when cells reached approximately 40%-50% confluence, the inserts containing RAW264.7 cells were transferred into wells seeded with mRTECs to initiate indirect co-culture. The co-culture system was maintained in low-glucose DMEM supplemented with 10% FBS and 1% penicillin/streptomycin. Cells were then subjected to H/R treatment. The experimental groups included mRTECs alone under H/R, mRTECs co-cultured with RAW264.7 cells under H/R, RAW264.7 cells alone under H/R, and RAW264.7 cells co-cultured with mRTECs under H/R. Where indicated, sc-BPNSs were added at 20 μg/mL before H/R induction and maintained during the co-culture period. After treatment, cells were collected separately for protein extraction and subsequent analyses.

### Statistical Analyses

All statistical analyses were performed using GraphPad Prism 9.0. Data are expressed as the mean ± standard deviation (SD). Normality and homogeneity of variance were assessed before statistical comparison. Differences between two groups were analyzed using an unpaired Student’s t-test. Differences among multiple groups were analyzed by one-way ANOVA followed by Tukey’s post hoc test when homogeneity of variance was met. When homogeneity of variance was not met, Welch’s ANOVA or appropriate nonparametric tests were used. Statistical significance was defined as *P < 0.05 and **P < 0.01; ns denotes no significant difference.

## Results

### Physicochemical properties, antioxidant capacity, and renal accumulation of sc-BPNSs

**Figure 1:**
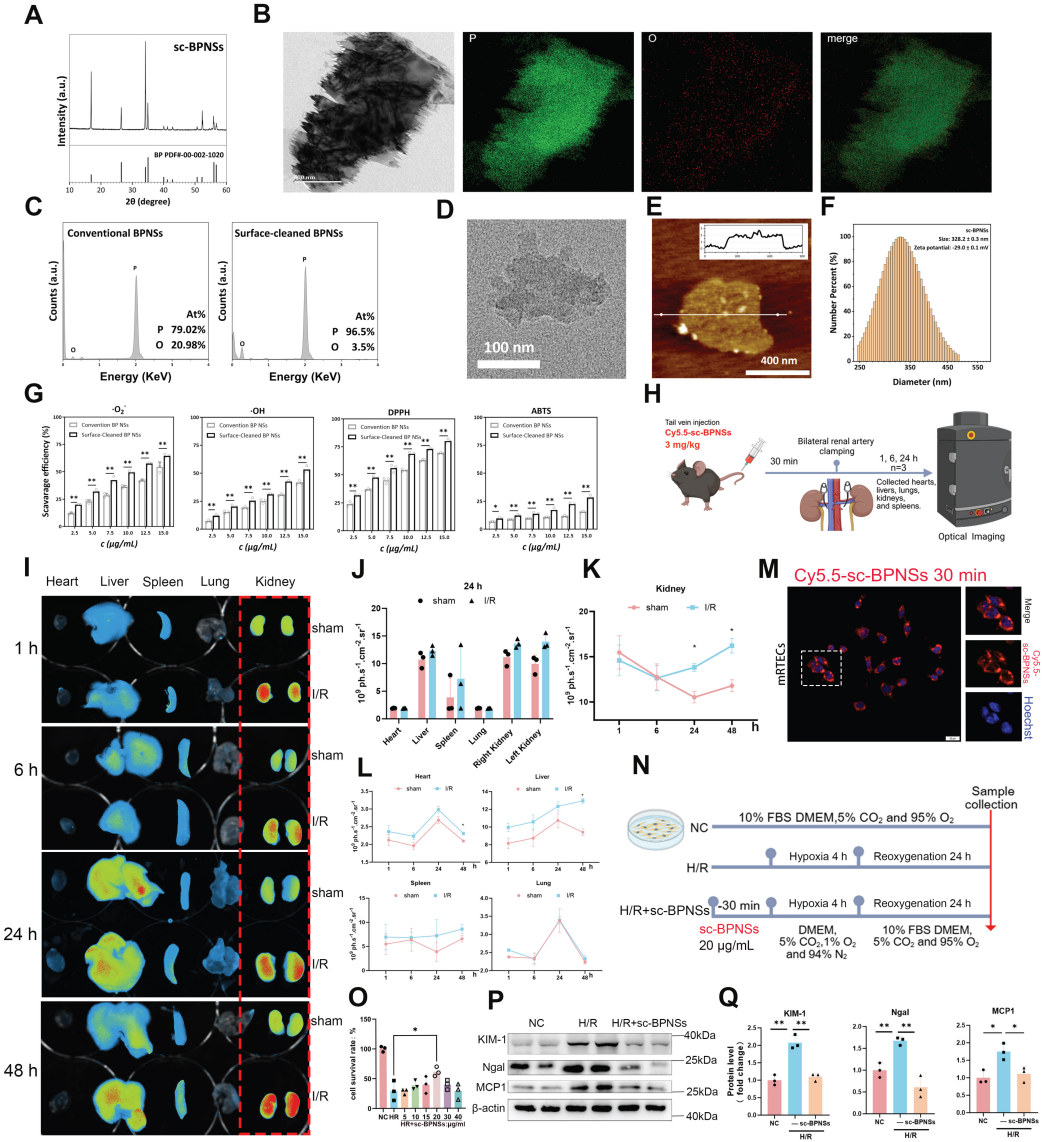
** Physicochemical properties antioxidant capacity and renal accumulation of sc-BPNSs.** (A) XRD pattern of sc-BPNSs. (B) TEM image of sc-BPNSs (left) and corresponding elemental mapping images showing phosphorus (P) and oxygen (O) distribution. (C) EDS analysis of BPNSs before and after surface modification. (D) High-resolution TEM image of sc-BPNSs. (E) AFM image and height profile, showing a nanosheet thickness of approximately 3 nm. (F) DLS profile of sc-BPNSs, showing particle size distribution. (G) Antioxidant capacity of sc-BPNSs measured by free radical scavenging assays for O₂⁻, •OH, DPPH, and ABTS radicals. Dose-dependent scavenging effects were observed (n = 3; *P < 0.05, **P < 0.01). (H) Schematic of the *in vivo* imaging workflow. Cy5.5-sc-BPNSs were administered via tail vein injection at 3 mg/kg, and organs were harvested at the indicated time points for fluorescence imaging. (I) Representative fluorescence images showing the distribution of Cy5.5-sc-BPNSs in major organs. (J) Quantification of Cy5.5 fluorescence intensity in major organs at 24 h after injection. Kidney fluorescence was significantly elevated in the I/R group. (K) Time-dependent changes in renal fluorescence intensity after Cy5.5-sc-BPNSs injection (n = 3; *P < 0.05). (L) Quantification of fluorescence intensity in the heart, liver, spleen, and lung. The liver and spleen showed moderate accumulation, whereas the heart and lung showed relatively low uptake. (M) Confocal microscopy showing intracellular uptake of Cy5.5-sc-BPNSs (red) by mRTECs. Nuclei were counterstained with Hoechst (blue). (N) Schematic of *in vitro* H/R injury model and sc-BPNS treatment protocol.(O) Cell viability of mRTECs treated with increasing concentrations of sc-BPNSs (5-40 μg/mL) under H/R conditions, as measured by CCK-8 assay. sc-BPNSs at 20 μg/mL significantly improved cell viability compared with H/R treatment alone (n = 3; *P < 0.05). (P) Representative Western blot bands of KIM-1, Ngal, and MCP-1 in mRTECs under different treatments. (Q) Quantitative analysis of KIM-1, Ngal, and MCP-1 protein expression levels (n = 3; *P < 0.05, **P < 0.01).

The physicochemical features of sc-BPNSs were first systematically characterized. X-ray diffraction (XRD) showed the characteristic diffraction peaks of crystalline black phosphorus, indicating that the black phosphorus lattice was preserved after hydrogen reduction (Figure 1A). TEM revealed a thin, layered nanosheet morphology, while elemental mapping showed strong phosphorus signals and only weak oxygen signals, consistent with markedly reduced surface oxidation (Figure 1B). Energy-dispersive X-ray spectroscopy (EDS) further confirmed this change. Compared with conventional BPNSs, the oxygen atomic percentage in sc-BPNSs decreased from 20.98% to 3.5%, whereas the phosphorus atomic percentage increased from 79.02% to 96.5% (Figure 1C), demonstrating efficient removal of surface oxygen species. High-resolution TEM (HRTEM) showed distinct lattice fringes, further supporting the preserved crystalline structure of sc-BPNSs (Figure 1D). Atomic force microscopy (AFM) confirmed the nanoscale thickness of the sheets (Figure 1E), and dynamic light scattering (DLS) showed a hydrodynamic diameter of approximately 328.2 ± 0.1 nm and a zeta potential of -29.0 ± 0.1 mV (Figure 1F). Overall, these results indicate the successful preparation of well-dispersed sc-BPNSs with substantially reduced surface oxygen content.

We next evaluated the colloidal properties of sc-BPNSs in PBS. Compared to conventional BPNSs, sc-BPNSs exhibited a more negative zeta potential, consistent with the surface chemical changes caused by hydrogen reduction (Figure S1B). The dispersion stability of sc-BPNSs was further assessed by monitoring their hydrodynamic diameter in PBS over 7 days. No obvious size change was observed during this period, indicating that sc-BPNSs maintained good colloidal stability under the tested conditions (Figure S1C).

The antioxidant capacity of sc-BPNSs was then examined using four radical-scavenging assays, including superoxide anion radical (O₂⁻), hydroxyl radical (•OH), 1,1-Diphenyl-2-picrylhydrazyl (DPPH), and 2,2'-Azinobis(3-ethylbenzothiazoline-6-sulfonic acid) diammonium salt (ABTS). In all assays, sc-BPNSs showed concentration-dependent radical-scavenging activity and consistently outperformed conventional BPNSs, supporting the notion that removal of surface oxidation enhances the intrinsic redox activity of BPNSs (Figure 1G).

Finally, the *in vivo* distribution of sc-BPNSs was examined using intravenously injected Cy5.5-sc-BPNSs, followed by *ex vivo* fluorescence imaging of major organs at different time points (Figure 1H-I). In a bilateral I/R model, Cy5.5-sc-BPNSs showed marked renal accumulation, with overall stronger kidney-associated fluorescence in I/R mice than in sham controls. Quantitative analysis at 24 h further confirmed higher signals in both kidneys of I/R mice, while detectable distribution was also observed in the liver and spleen (Figure 1J). Time-course analysis showed that renal fluorescence remained elevated in I/R mice and was still higher than that in sham mice at later time points (Figure 1K). Dynamic signal changes were also observed in the heart, liver, spleen, and lung (Figure 1L). These findings indicate that sc-BPNSs accumulate prominently in the kidney under AKI conditions.

To further assess accumulation associated with renal injury, a unilateral left renal I/R model was established (Figure S1D). Whole-body imaging of ICG-sc-BPNSs showed limited anatomical resolution, with only weak signals detected over the renal region from the dorsal view (Figure S1E). Therefore, Cy5.5-sc-BPNSs were used for* ex vivo* kidney imaging. Injured kidneys showed stronger fluorescence than sham kidneys at 1, 6, and 24 h, and quantitative analysis confirmed significantly higher signals in I/R kidneys (Figure S1F and G). These results are consistent with preferential retention of sc-BPNSs in injured renal tissue.

Cellular uptake of sc-BPNSs was then evaluated in mRTECs. After incubation with Cy5.5-sc-BPNSs for 30 min, confocal microscopy showed prominent red fluorescence within the cytoplasm, with minimal overlap with Hoechst-stained nuclei. This distribution pattern suggests that sc-BPNSs were rapidly internalized by mRTECs and were predominantly distributed in the cytoplasmic compartment (Figure 1M).

To determine whether cellular uptake was associated with cytoprotection, an *in vitro* H/R model was established to mimic AKI-related tubular injury (Figure 1N). Cell viability assay showed that sc-BPNS treatment partially restored mRTEC survival under H/R conditions (Figure 1O). Consistently, western blot analysis demonstrated that H/R increased the kidney injury-associated proteins kidney injury molecule-1 (KIM-1), neutrophil gelatinase-associated lipocalin (Ngal), and monocyte chemoattractant protein-1 (MCP-1), whereas pretreatment with sc-BPNSs reduced their induction (Figure 1P and Q). These results indicate that sc-BPNSs confer direct protection against tubular epithelial injury both *in vivo* and in cultured mRTECs exposed to H/R.

We further assessed the subacute toxicity of sc-BPNSs *in vivo*. C57BL/6 mice were injected with sc-BPNSs via the tail vein at 3 mg/kg every 3 days for 28 days, while control mice received the same volume of saline. Serum biochemical analysis showed no significant differences in AST, AST/ALT ratio, CK-MB, sCr between the two groups (Figure S1I-L). ALT was mildly increased in the sc-BPNS-treated group (Figure S1H), but the value remained within the physiological range 23. H&E staining showed no apparent histopathological abnormalities in the heart, liver, spleen, lung, or kidney (Figure S1M). Thus, repeated administration of sc-BPNSs did not cause obvious subacute systemic toxicity under the tested conditions.

### sc-BPNSs attenuate AKI and oxidative stress

**Figure 2:**
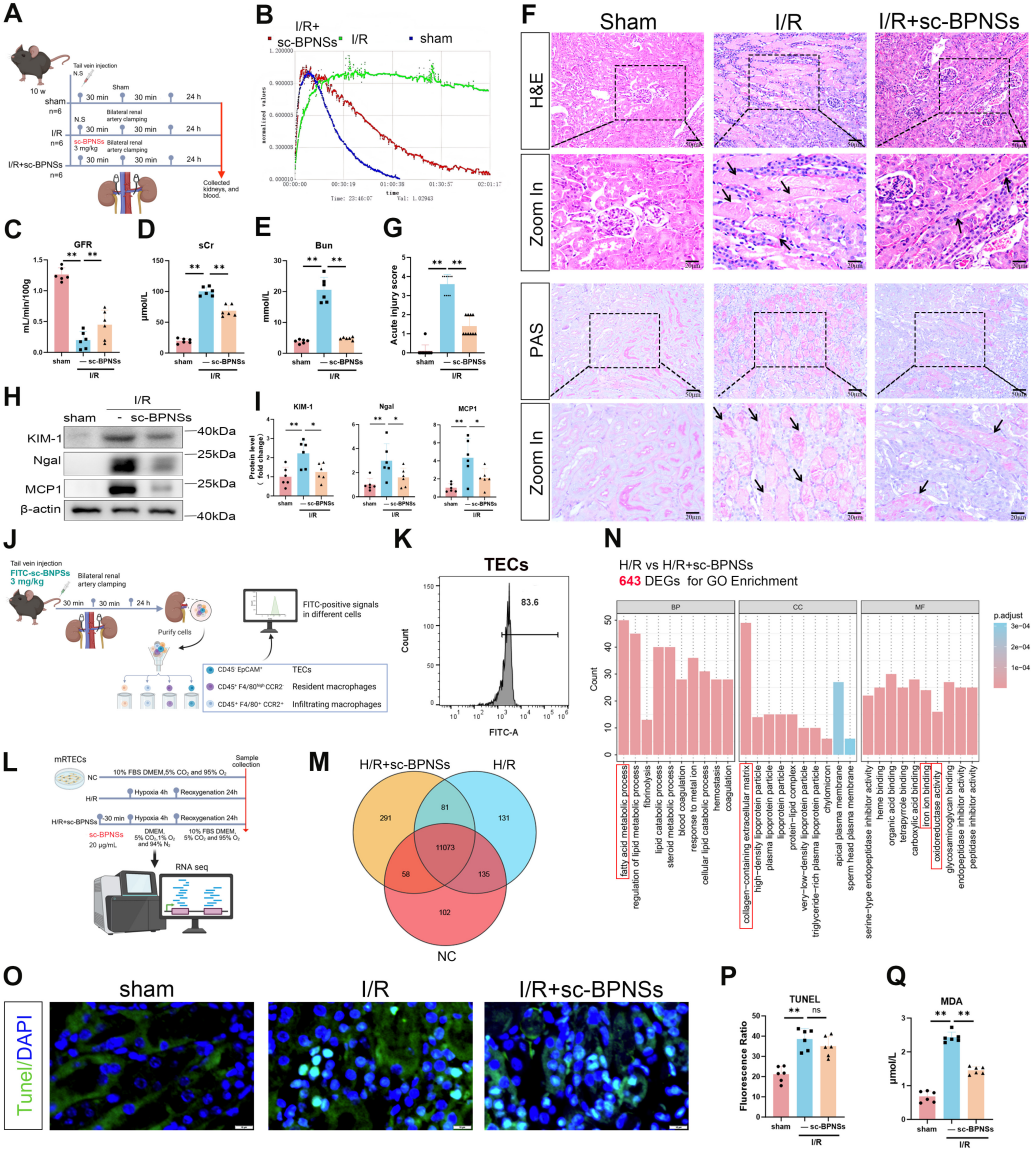
** sc-BPNSs improve renal function, tissue structure, and oxidative stress in the I/R-induced AKI model.** (A) Schematic diagram of the* in vivo* I/R injury model and sc-BPNS intervention protocol. (B) GFR monitoring using FITC-sinistrin-based transdermal fluorescence decay curves. (C) Quantification of GFR (n = 6; *P < 0.05, **P < 0.01). (D) sCr levels (n = 6; **P < 0.01). (E) BUN levels (n = 6; **P < 0.01). (F) Representative H&E and PAS staining images of kidney sections from each group, with corresponding magnified views. Arrows indicate infarct-like injured areas. (G) Tubular injury scores quantified from H&E-stained sections (n = 10 fields per group; **P < 0.01). (H) Representative Western blot bands of KIM-1, Ngal, and MCP-1 in renal tissue from different groups. (I) Quantification of KIM-1, Ngal, and MCP-1 protein expression levels in renal tissue (n = 6; *P < 0.05, **P < 0.01). (J) Schematic illustration of the *in vivo* cellular uptake assay. Mice were injected intravenously with FITC-sc-BPNSs at 3 mg/kg, subjected to bilateral I/R, and kidneys were harvested 24 h later for single-cell suspensions preparation. Different renal cell populations were identified by flow cytometry, including CD45⁻EpCAM⁺ tubular epithelial cells, CD45⁺F4/80^high^CCR2⁻ resident macrophages, and CD45⁺F4/80⁺CCR2⁺ infiltrating macrophages, followed by detection of FITC signals in each population. (K) Representative flow cytometric analysis of FITC signals in tubular epithelial cells, showing a distinct FITC-positive population after FITC-sc-BPNS administration. (L) Workflow diagram of RNA sequencing analysis in the H/R cell model. (M) Venn diagram showing the overlap of DEGs among comparison groups.(N) GO enrichment analysis of DEGs. (O) Representative TUNEL staining images of renal sections. TUNEL-positive apoptotic cells are shown in green, and nuclei are counterstained with DAPI in blue. (P) Quantification of TUNEL-positive fluorescence intensity (n = 6; **P < 0.01; ns, not significant). (Q) MDA levels in renal tissue, used as an index of lipid peroxidation (n = 6; **P < 0.01).

To evaluate the therapeutic efficacy of sc-BPNSs in I/R-induced AKI, a bilateral renal I/R model was established in mice (Figure 2A). Glomerular filtration function was assessed using transdermal FITC-sinistrin clearance monitoring, which showed that I/R injury markedly delayed tracer elimination, whereas sc-BPNS treatment partially improved this abnormality (Figure 2B). Quantitative analysis of the clearance curves further demonstrated that the glomerular filtration rate (GFR) was significantly reduced in the I/R group and markedly restored by sc-BPNS treatment (Figure 2C). In parallel, sCr and BUN levels were significantly elevated after I/R injury, and both were attenuated by sc-BPNS administration (Figure 2D and E).

Histological analysis further confirmed the renoprotective effect of sc-BPNSs. H&E and PAS staining revealed prominent tubular dilation, epithelial injury, and focal infarct-like lesions in the I/R group, with arrows indicating injured regions. PAS staining highlighted brush border loss, tubular epithelial desquamation, and disruption of tubular architecture. These pathological alterations were markedly alleviated after sc-BPNS treatment (Figure 2F). Semiquantitative acute injury scoring further supported these histological findings (Figure 2G). Western blot analysis showed that I/R markedly increased the expression of the kidney injury-associated proteins KIM-1 and Ngal, as well as the inflammatory chemokine MCP-1, whereas sc-BPNS treatment substantially suppressed their upregulation (Figure 2H and I).

To further evaluate whether sc-BPNSs exert protective effects in other forms of AKI, Cis- and FA-induced AKI mouse models were established. In the Cis model, sc-BPNSs significantly reduced Cis-induced increases in sCr and BUN, alleviated tubular injury and acute injury scores, and suppressed the upregulation of KIM-1, Ngal, and MCP-1 (Figure S2A-I). Similarly, in the FA model, sc-BPNSs markedly improved renal dysfunction, reduced histopathological injury, and inhibited the increased expression of KIM-1, Ngal, and MCP-1 induced by FA treatment (Figure S2J-R). These findings support a broad renoprotective effect of sc-BPNSs across AKI models with different etiologies.

To further define the cellular basis of this protection, uptake of sc-BPNSs by renal cell populations was examined *in vivo*. FITC-sc-BPNSs were intravenously administered to mice subjected to bilateral renal I/R, followed by flow cytometric analysis of kidney single-cell suspensions at 24 h (Figure 2J). A prominent FITC-positive population was detected among CD45^-^EpCAM^+^ tubular epithelial cells, with a positive rate of 83.6%, indicating efficient uptake of sc-BPNSs by renal tubular epithelial cells *in vivo* (Figure 2K).

To further explore the underlying mechanism, RNA sequencing was performed in mRTECs subjected to H/R in the presence or absence of sc-BPNSs (Figure 2L). Correlation analysis showed good intragroup reproducibility among samples (Figure S3A). Venn diagram analysis identified 11,073 commonly expressed genes across the three groups (Figure 2M). Differentially expressed genes (DEGs) between the H/R and H/R + sc-BPNSs groups indicated that sc-BPNSs markedly remodeled the H/R-induced transcriptional program (Figure S3B). A total of 643 DEGs between the H/R and H/R + sc-BPNSs groups were subjected to Gene Ontology (GO) analysis (Figure 2N). These DEGs were mainly enriched in biological processes and functions related to lipid metabolism, collagen-containing extracellular matrix, iron ion binding, and antioxidant activity. Kyoto Encyclopedia of Genes and Genomes (KEGG) analysis further revealed significant enrichment of oxidative stress-related pathways, including arachidonic acid metabolism and peroxisome signaling (Figure S3C).

Despite these protective effects, TUNEL staining showed that sc-BPNS treatment did not significantly reduce apoptosis in I/R kidneys (Figure 2O and P). In contrast, MDA measurement showed that sc-BPNSs significantly reduced I/R-induced lipid peroxidation (Figure 2Q). Taken together, these findings suggest that the renoprotective effect of sc-BPNSs is more closely associated with attenuation of lipid peroxidation and oxidative injury than with robust suppression of apoptosis. Moreover, the enrichment of genes associated with oxidative stress, fatty acid metabolism, and iron handling, together with the observed reduction in lipid peroxidation, supports the possibility that sc-BPNSs mitigate tubular injury at least in part through suppression of ferroptosis.

### sc-BPNSs inhibit ferroptosis during AKI by restoring GPX4 and FTH1 and reducing lipid peroxidation

**Figure 3:**
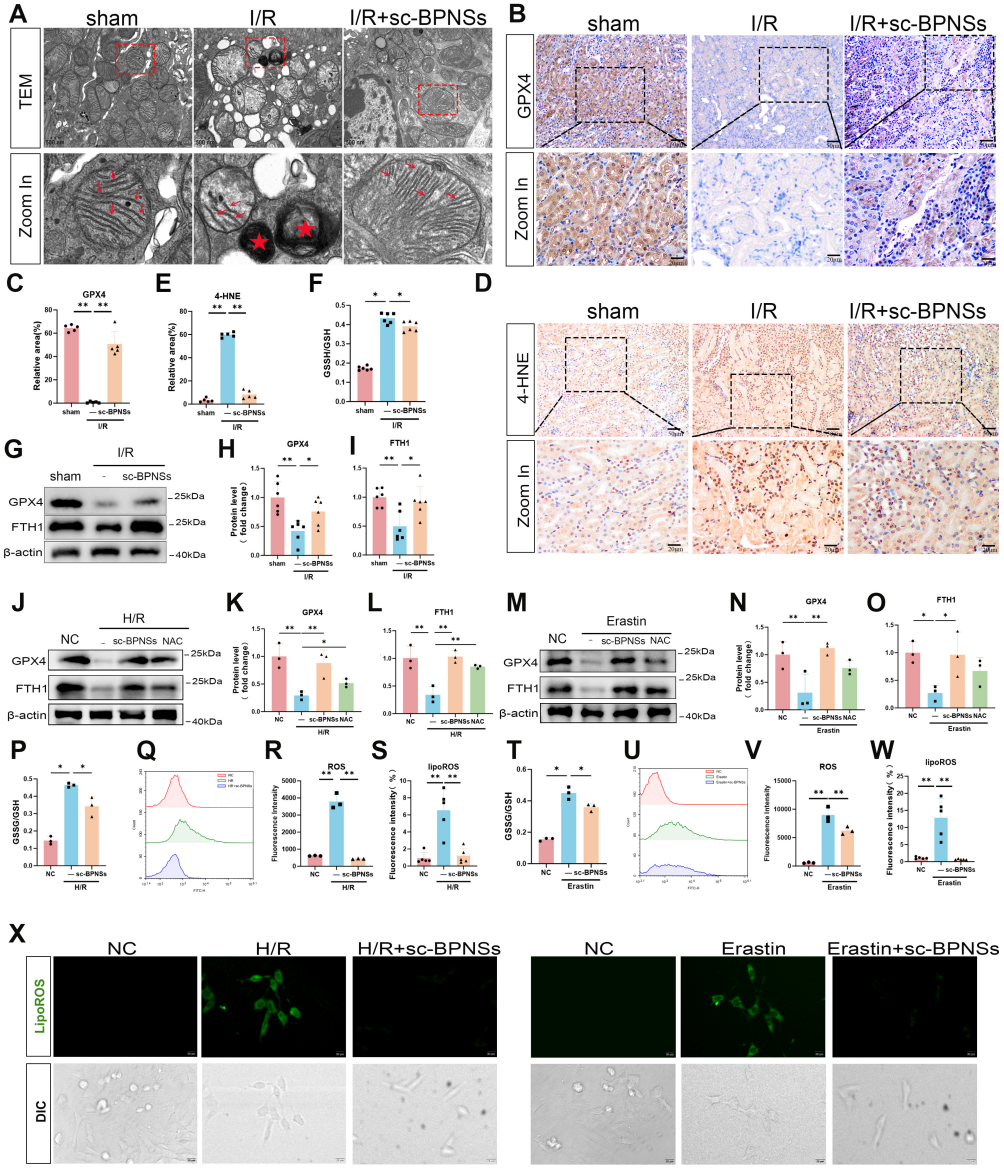
** sc-BPNSs inhibit ferroptosis during AKI by restoring GPX4 and FTH1 and reducing lipid peroxidation.** (A) TEM images showing ferroptosis-associated mitochondrial ultrastructural changes in renal tubular epithelial cells from sham, I/R, and I/R+sc-BPNSs groups. Red arrows indicate mitochondrial cristae, and red asterisks indicate disrupted mitochondrial membranes. (B) Immunohistochemical staining of GPX4 in kidney tissues from sham, I/R, and I/R + sc-BPNSs groups. (C) Quantification of GPX4-positive areas in kidney sections (n = 6; *P < 0.05, **P < 0.01). (D) Immunohistochemical staining of 4-HNE in kidney tissues from sham, I/R, and I/R + sc-BPNSs groups. (E) Quantification of 4-HNE-positive areas in kidney sections (n = 5; **P < 0.01). (F) GSSG/GSH ratio in kidney tissues (n = 6; *P < 0.05). (G) Representative Western blot analysis of GPX4 and FTH1 protein expression in kidney tissues. (H, I) Quantitative analysis of GPX4 and FTH1 protein levels shown in panel G (n = 6; *P < 0.05, **P < 0.01). (J) Representative Western blot analysis of GPX4 and FTH1 in mRTECs subjected to H/R in the presence or absence of sc-BPNSs or NAC (2 mM). (K-L) Quantitative analysis of GPX4 and FTH1 protein levels shown in panel J (n = 3; *P < 0.05, **P < 0.01). (M) Representative Western blot analysis of GPX4 and FTH1 in mRTECs treated with erastin in the presence or absence of sc-BPNSs or NAC (2 mM). (N, O) Quantitative analysis of GPX4 and FTH1 protein levels shown in panel M (n = 3; *P < 0.05, **P < 0.01). (P) GSSG/GSH ratio in mRTECs under H/R conditions (n = 3; *P < 0.05). (Q) Representative flow cytometry plots showing intracellular ROS levels in mRTECs under H/R conditions. (R) Quantification of intracellular ROS levels shown in panel Q (n = 3; **P < 0.01). (S) Quantification of lipid ROS fluorescence intensity in mRTECs under H/R conditions (n = 3; **P < 0.01). (T) GSSG/GSH ratio in mRTECs treated with erastin (n = 3; *P < 0.05). (U) Representative flow cytometry plots showing intracellular ROS levels in mRTECs treated with erastin. (V) Quantification of intracellular ROS levels shown in panel (U) (n = 3; **P < 0.01). (W) Quantification of lipid ROS fluorescence intensity in mRTECs treated with erastin (n = 3; **P < 0.01). (X) Representative Liperfluo fluorescence images showing lipid ROS accumulation in mRTECs under H/R or erastin treatment.

Renal tubular mitochondrial ultrastructure was examined by TEM to determine whether ferroptosis-related injury contributed to the renoprotective effects of sc-BPNSs. In the I/R group, tubular epithelial cells showed typical ferroptosis-associated mitochondrial changes, including loss or marked reduction of cristae, increased membrane density, and outer membrane rupture. These mitochondrial abnormalities were less pronounced after sc-BPNS treatment (Figure 3A).

GPX4 was then examined in kidney sections. Immunohistochemical staining showed that GPX4 expression declined markedly after I/R injury and was partly recovered after sc-BPNS treatment (Figure 3B and C). Because GPX4 detoxifies lipid peroxides, 4-hydroxynonenal (4-HNE) staining was used to assess lipid peroxidation in renal tissue. 4-HNE staining was strongly enhanced in the I/R group, whereas sc-BPNS treatment clearly reduced this signal (Figure 3D and E). The oxidized glutathione/reduced glutathione (GSSG/GSH) ratio was also significantly increased after I/R injury and decreased following sc-BPNS administration (Figure 3F). Western blotting further showed that I/R reduced GPX4 and ferritin heavy chain 1 (FTH1) protein levels, while sc-BPNSs restored both proteins (Figure 3G-I). Taken together, the mitochondrial, histological, biochemical, and protein data support that sc-BPNSs reduce I/R-induced lipid peroxidation and ferroptosis-associated tubular injury *in vivo*.

To further validate this effect *in vitro*, H/R and erastin-induced ferroptosis models were established in mRTECs, with N-acetylcysteine (NAC), a well-established antioxidant that replenishes intracellular glutathione, included as a positive control. In the H/R model, both sc-BPNSs and NAC restored the expression of GPX4 and FTH1, and sc-BPNSs markedly reversed the H/R-induced downregulation of these proteins (Figure 3J-L). Consistently, the elevated GSSG/GSH ratio induced by H/R was significantly reduced by sc-BPNSs (Figure 3P). Flow cytometric analysis further showed that H/R markedly increased intracellular ROS accumulation, which was significantly attenuated by sc-BPNSs (Figure 3Q and R). Liperfluo staining showed that lipid ROS levels were markedly elevated under H/R conditions and substantially reduced by sc-BPNS treatment, as further supported by representative fluorescence images (Figure 3S and X).

Similar results were obtained in the erastin model, a classical chemical model of ferroptosis. Erastin treatment markedly reduced GPX4 and FTH1 expression, whereas sc-BPNSs partially restored both proteins. NAC also partially reversed the erastin-induced reduction in GPX4 and showed a protective trend toward FTH1 expression (Figure 3M-O). Consistently, the elevated GSSG/GSH ratio induced by erastin was significantly reduced by sc-BPNSs (Figure 3T). Flow cytometric analysis further showed that erastin markedly increased intracellular ROS accumulation, which was significantly attenuated by sc-BPNSs (Figure 3U and V). Liperfluo staining showed that lipid ROS levels were markedly elevated after erastin treatment and substantially reduced by sc-BPNSs, as further supported by the representative fluorescence images (Figure 3W and X).

Taken together, these data from kidney tissues and mRTECs demonstrate that sc-BPNSs effectively attenuate ferroptosis under I/R-, H/R-, and erastin-induced injury conditions. The protective effects of sc-BPNSs were consistently associated with restoration of GPX4 and FTH1, suppression of oxidative and lipid peroxidative stress, and preservation of mitochondrial structure. These results suggest that inhibition of ferroptosis is an important contributor to the renal protection afforded by sc-BPNSs.

### sc-BPNSs suppress ferroptosis through regulation of TFR2-associated iron homeostasis

**Figure 4:**
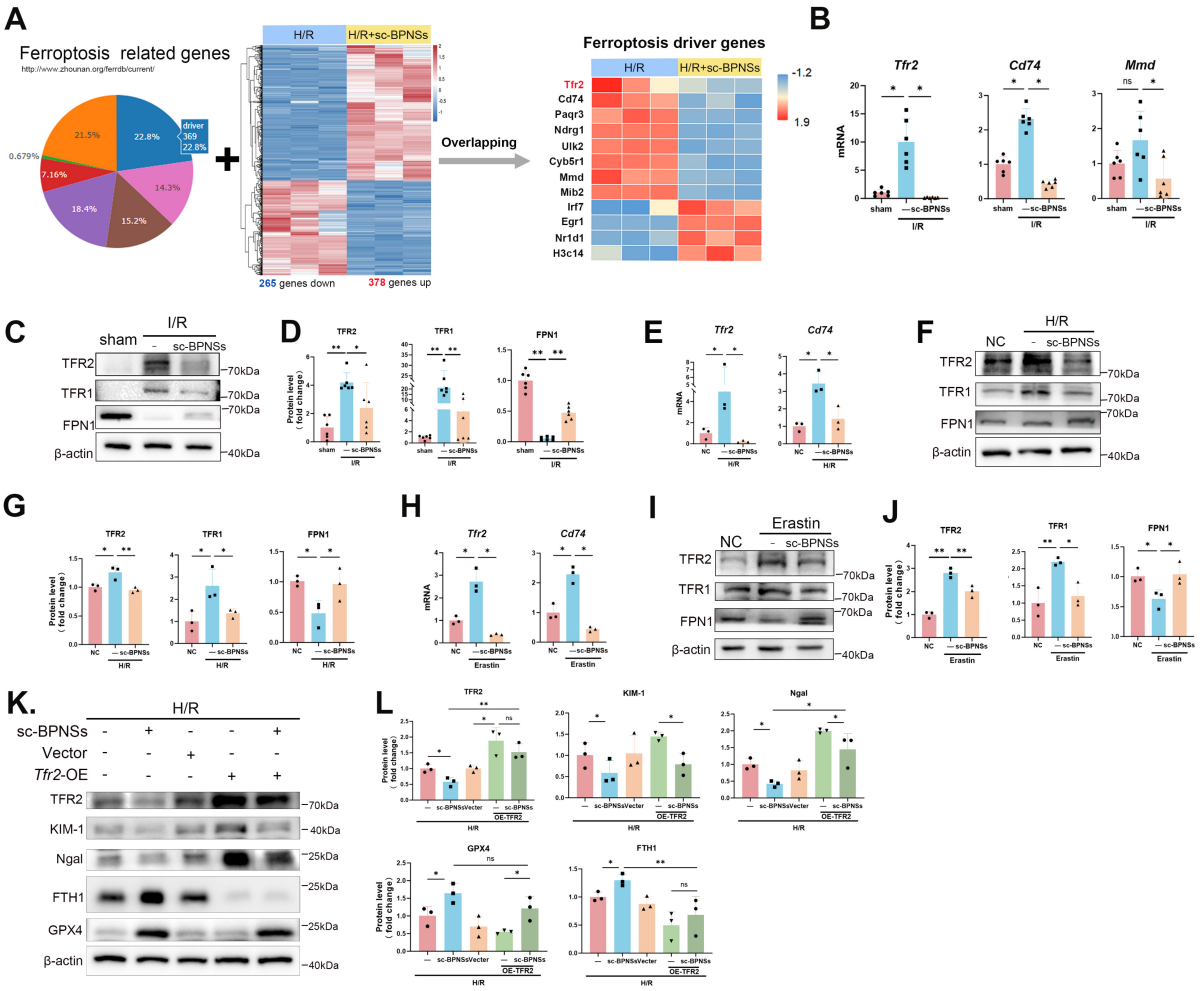
** sc-BPNSs inhibit ferroptosis and tubular injury by downregulating TFR2 expression.** (A) Left: Pie chart showing the functional classification of ferroptosis-related genes, including drivers (22.8%), suppressors (21.5%), inducers (18.4%), inhibitors (15.2%), disease-associated genes (14.3%), unclassified genes (7.16%), and markers (0.679%). Middle: Heatmap of RNA-seq-derived DEGs between the H/R and H/R + sc-BPNSs groups, showing 265 downregulated and 378 upregulated genes. Right: Venn diagram showing the intersection of these DEGs with ferroptosis driver genes listed in the FerrDb database. *Tfr2*, *Cd74*, and *Mmd* were selected as candidate targets. (B) qPCR analysis of *Tfr2*, *Cd74*, and *Mmd* mRNA expression in I/R kidney tissues (n = 6; *P < 0.05; ns, not significant). (C) Representative Western blot analysis of TFR2, TFR1, and FPN1 protein expression in renal tissues from different groups. (D) Quantification of TFR2, TFR1, and FPN1 protein levels* in vivo* (n = 6; *P < 0.05, **P < 0.01). (E) Relative mRNA expression of *Tfr2* and *Cd74* in mRTECs under H/R conditions (n = 3; *P < 0.05). (F) Representative Western blot analysis of TFR2, TFR1, and FPN1 in H/R-treated cells. (G) Quantitative analysis of TFR2, TFR1, and FPN1 protein expression *in vitro* (n = 3; *P < 0.05, **P < 0.01). (H) mRNA expression of *Tfr2* and *Cd74* under erastin-induced ferroptosis (n = 3; *P < 0.05). (I) Representative Western blot analysis of TFR2, TFR1, and FPN1 under erastin treatment. (J) Quantification of TFR2, TFR1, and FPN1 protein levels in erastin-treated cells (n = 3; *P < 0.05, **P < 0.01). (K) Representative Western blot analysis of TFR2, GPX4, FTH1, KIM-1, and Ngal in H/R-treated cells with *Tfr2* overexpression and/or sc-BPNS treatment. (L) Quantification of TFR2, GPX4, FTH1, KIM-1, and Ngal protein expression in H/R-treated cells following TFR2 overexpression and/or sc-BPNS treatment (n = 3; *P < 0.05, **P < 0.01; ns, not significant).

To further elucidate how sc-BPNSs modulate ferroptosis, DEGs after sc-BPNS treatment were intersected with ferroptosis driver genes curated in FerrDb, leading to the identification of transferrin receptor 2 (*Tfr2*)*,* cluster of differentiation 74 (*Cd74*), and monocyte to macrophage differentiation-associated (*Mmd*) as ferroptosis-associated candidates enriched in the dataset (Figure 4A). In I/R kidneys, qPCR showed that *Tfr2* and *Cd74* were significantly upregulated and markedly reduced by sc-BPNSs, whereas *Mmd* showed a nonsignificant increasing trend that was also suppressed by sc-BPNSs (Figure 4B). Consistently, Western blot analysis demonstrated that sc-BPNSs reduced the expression of the iron uptake proteins TFR2 and transferrin receptor 1 (TFR1), while increasing the expression of the iron export protein ferroportin (FPN1) in kidney tissue (Figure 4C and D).

Similar patterns were observed in both H/R- and erastin-treated mRTECs. In the H/R model, sc-BPNSs reduced the mRNA expression of *Tfr2* and *Cd74,* and suppressed TFR2/TFR1 protein expression while enhancing FPN1 expression (Figure 4E-G). Although *Mmd* was not significantly induced by H/R, its expression decreased after sc-BPNS treatment (Figure S3D). In the erastin model, sc-BPNSs again reduced *Tfr2* and *Cd74* mRNA expression and shifted iron-handling protein expression toward decreased TFR2/TFR1 and increased FPN1 (Figure 4H-J), whereas *Mmd* expression remained unchanged (Figure S3E). Together, these findings indicate that sc-BPNSs consistently suppress iron uptake-associated programs and promote iron export across both *in vivo* and *in vitro* ferroptotic injury settings.

To validate the functional relevance of TFR2 in ferroptosis modulation, *Tfr2* overexpression was established in the H/R model. *Tfr2* overexpression further aggravated tubular injury, as shown by reduced GPX4 expression and increased KIM-1 and Ngal levels. sc-BPNSs partially restored GPX4 expression and suppressed the upregulation of KIM-1 and Ngal, even in *Tfr2*-overexpressing cells (Figure 4L). In contrast, FTH1 expression was not fully recovered, suggesting that the protective effect of sc-BPNSs is not mediated solely through ferritin-related iron storage. One plausible explanation is that sc-BPNSs compensate, at least in part, for incomplete FTH1 restoration by directly binding bioavailable iron and reducing the labile iron pool. This interpretation is supported by previous evidence showing that black phosphorus can coordinate with Fe³⁺ through charge transfer, providing a potential chemical basis for the iron-buffering activity of sc-BPNSs 24.

### sc-BPNSs directly chelate iron and alleviate iron overload-associated injury

**Figure 5:**
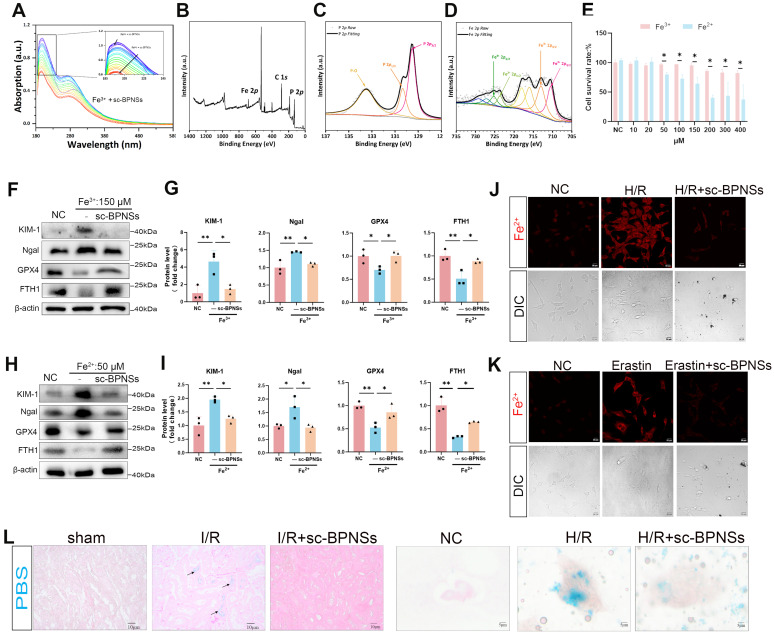
** sc-BPNSs inhibit ferroptosis by chelating iron ions and regulating iron homeostasis.** (A) UV-Vis absorption spectra showing a blue shift and decreased absorbance of Fe³⁺ solution upon gradual addition of sc-BPNSs, indicating sc-BPNSs-Fe³⁺ binding. (B) XPS full survey spectrum of the precipitate after sc-BPNSs-Fe³⁺ titration, revealing coexistence of Fe and P elements. (C) High-resolution P 2p XPS spectra showing P-O and P-metal bonds, with a positive shift in P-metal binding energy suggesting electron transfer from P to Fe³⁺. (D) Fe 2p XPS spectra indicating partial reduction of Fe³⁺ to Fe²⁺ by sc-BPNSs. (E) CCK-8 assay assessing cell viability under different concentrations of Fe²⁺ and Fe³⁺ (n = 3; **P < 0.01). (F) Representative Western blot analysis of KIM-1, Ngal, GPX4, and FTH1 in cells under Fe³⁺-induced ferroptosis. (G) Quantification of KIM-1, Ngal, GPX4, and FTH1 protein levels in the Fe³⁺ model (n = 3; *P < 0.05, **P < 0.01). (H) Representative Western blot analysis of KIM-1, Ngal, GPX4, and FTH1 in cells treated with Fe²⁺. (I) Quantitative analysis of KIM-1, Ngal, GPX4, and FTH1 protein expression in the Fe²⁺ model (n = 3; *P < 0.05, **P < 0.01). (J) Detection of intracellular Fe²⁺ using fluorescent probes under H/R treatment, showing reduced Fe²⁺ accumulation after sc-BPNS treatment. (K) Detection of intracellular Fe²⁺ using fluorescent probes under erastin treatment, showing reduced Fe²⁺ accumulation after sc-BPNS treatment. (L) Prussian blue staining of kidney tissues and mRTECs, revealing reduced Fe³⁺ deposition in sc-BPNS-treated I/R and H/R models.

Given the observed restoration of iron homeostasis and suppression of ferroptosis by sc-BPNSs, direct iron chelation was next investigated as a potential underlying mechanism. Ultraviolet-visible (UV-Vis) spectroscopy was first performed to assess the interaction between sc-BPNSs and Fe³⁺ in solution by titration. Upon addition of sc-BPNSs to the Fe³⁺ solution, the characteristic absorption peak of Fe³⁺ at 291 nm exhibited a blue shift, and absorbance intensity progressively decreased with increasing sc-BPNS concentration, indicating effective binding and depletion of free Fe³⁺ by sc-BPNSs (Figure 5A). X-ray photoelectron spectroscopy (XPS) confirmed the formation of sc-BPNS-Fe³⁺ complexes, as shown by the simultaneous detection of P and Fe signals (Figure 5B). Deconvolution of the P *2p* spectrum identified P-O, P-metal, and P-P components, and the positive shift of the P-metal peak suggested electron transfer from phosphorus to Fe³⁺ (Figure 5C). In the Fe *2p* spectrum, signals corresponding to Fe²⁺ and Fe³⁺ appeared at 709.8 eV and 712.4 eV, respectively, suggesting that part of Fe³⁺ was reduced to Fe²⁺ after interaction with sc-BPNSs (Figure 5D). This result agrees with the redox interaction inferred from the P-metal shift. Fe³⁺-bound sc-BPNSs (Fe³⁺/sc-BPNSs) were also tested in the DPPH radical scavenging assay. Fe³⁺/sc-BPNSs still showed DPPH radical-scavenging activity after Fe³⁺ binding (Figure S3F). Overall, these data show that sc-BPNSs can capture iron in aqueous solution, form stable iron-containing complexes, and retain antioxidant activity after Fe³⁺ coordination. Surface cleaning of BPNSs may expose more phosphorus atoms carrying lone-pair electrons, which could strengthen the interaction between BPNSs and Fe³⁺ and make the iron-chelating behavior more detectable.

The functional consequences of this iron-binding activity were next examined by establishing Fe²⁺ and Fe³⁺ overload models in mRTECs. CCK-8 assays showed that 50 μM Fe²⁺ significantly reduced cell viability, whereas Fe³⁺ had no significant cytotoxic effect up to 150 μM (Figure 5E). Western blot analysis confirmed that 150 μM Fe³⁺ reduced GPX4 expression (Figure S3G), and this concentration was therefore used for subsequent Fe³⁺-induced ferroptosis experiments. In both Fe²⁺- and Fe³⁺-treated cells, sc-BPNSs restored GPX4 and FTH1 expression while reducing the injury markers KIM-1 and Ngal (Figure 5F-I), indicating protection against iron overload associated injury.

To determine whether sc-BPNSs also directly reduce intracellular iron accumulation, iron levels were next examined at the cellular and tissue levels. Fe²⁺-specific fluorescent probes showed that sc-BPNSs significantly decreased intracellular Fe²⁺ signals in both H/R- and erastin-treated cells (Figure 5J and K; quantification in Figure S3H and I). Prussian blue staining further demonstrated reduced Fe³⁺ deposition in both I/R kidney tissues and H/R-treated mRTECs after sc-BPNS treatment (Figure 5L). These findings support the view that sc-BPNSs directly chelate iron and alleviate iron overload-associated injury by reducing the bioavailability of redox active iron.

### sc-BPNSs modulate iron homeostasis proteins with IRP1- and STAT3-associated changes

**Figure 6:**
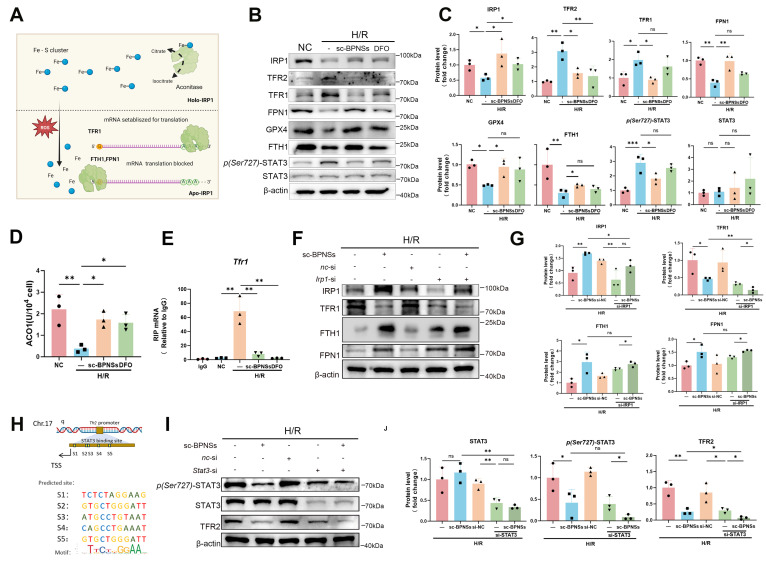
** sc-BPNSs modulate iron homeostasis proteins with IRP1- and STAT3-associated changes.** (A) Schematic illustration of IRP1 function. Oxidative stress damages Fe-S clusters, leading to Apo-IRP1 binding to IREs. (B) Representative Western blot analysis of IRP1, TFR2, TFR1, FPN1, GPX4, FTH1, *p*-STAT3 (Ser727), and total STAT3 in mRTECs subjected to H/R in the presence or absence of sc-BPNSs or DFO. (C) Quantitative analysis of the protein levels shown in panel B (n = 3; *P < 0.05, **P < 0.01; ns, not significant). (D) ACO1 activity in mRTECs subjected to H/R in the presence or absence of sc-BPNSs or DFO. (E) RIP analysis of IRP1 binding to *Tfr1* mRNA in mRTECs subjected to H/R in the presence or absence of sc-BPNSs or DFO. (F) Representative Western blot analysis of IRP1, TFR1, FTH1, and FPN1 in mRTECs subjected to H/R after siRNA-mediated IRP1 knockdown, with or without sc-BPNS treatment. (G) Quantitative analysis of the protein levels shown in panel F (n = 3; *P < 0.05, **P < 0.01; ns, not significant). (H) Predicted STAT3-binding sites in the *Tfr2* promoter based on JASPAR analysis. (I) Representative Western blot analysis of *p*-STAT3 (Ser727), total STAT3, and TFR2 in mRTECs subjected to H/R after siRNA-mediated STAT3 knockdown, with or without sc-BPNS treatment. (J) Quantitative analysis of the protein levels shown in panel I (n = 3; *P < 0.05, **P < 0.01; ns, not significant).

Having established that sc-BPNSs directly chelate iron and reduce intracellular iron burden, the effects of sc-BPNSs on the regulation of iron homeostasis proteins were next examined. Iron regulatory protein 1 (IRP1), also known as aconitase 1 (ACO1), is a central post-transcriptional regulator in the iron-responsive element-iron regulatory protein 1 (IRE-IRP1) iron-sensing system. The IRP1-associated regulatory branch was therefore first investigated. Under iron-replete conditions, IRP1 contains a [4Fe-4S] cluster and functions as cytosolic aconitase 1, referred to as Holo-IRP1. Under oxidative stress, this cluster is lost, and IRP1 is converted into its RNA-binding Apo form, which interacts with iron-responsive elements located in the untranslated regions of target mRNAs (Figure 6A). In its apo form, IRP1 binds IREs in target mRNAs, stabilizing *Tfr1* mRNA through its 3′ UTR and repressing *Fth1* and* Fpn1* translation through their 5′ UTRs. This regulatory pattern promotes iron uptake while limiting iron storage and export 25, 26.

Western blot analysis showed that H/R reduced the expression of IRP1, GPX4, FTH1, and FPN1, while increasing TFR1 expression. These changes were partially reversed by both sc-BPNSs and DFO, a classical iron chelator used here as a positive control, with sc-BPNSs showing a stronger effect (Figure 6B and C). Consistently, ACO1 activity was markedly suppressed by H/R, whereas both sc-BPNSs and DFO restored this activity (Figure 6D). RIP assays further showed that H/R enhanced the binding of IRP1 to *Tfr1* mRNA, and this interaction was attenuated by both sc-BPNSs and DFO (Figure 6E). In contrast, the binding signals of IRP1 to *Fth1* and *Fpn1* mRNAs were weak and showed no significant differences among groups (Figure S3J and K).

IRP1 knockdown experiments were next performed to determine whether the effects of sc-BPNSs on iron homeostasis-related proteins were dependent on the IRP1 pathway. IRP1 silencing reduced TFR1 expression, whereas FTH1 and FPN1 remained largely unchanged. Notably, after IRP1 knockdown, sc-BPNSs still partially restored IRP1 protein expression and further decreased TFR1 while increasing FTH1 and FPN1 (Figure 6F and G). These findings suggest that the effects of sc-BPNSs on iron homeostasis proteins are not fully dependent on IRP1 function alone and may involve preservation of IRP1 protein abundance.

Together, these findings support the involvement of the IRE-IRP1 regulatory effects in sc-BPNS-mediated modulation of iron homeostasis. However, because *Tfr2* mRNA lacks a canonical IRE motif, its regulation is unlikely to be explained by this mechanism alone. A possible transcriptional pathway governing TFR2 expression was therefore examined. JASPAR analysis identified multiple putative STAT3-binding sites within the *Tfr2* promoter region (Figure 6H). Consistent with this prediction, Western blot analysis showed that H/R increased *p*-STAT3 (Ser727) and TFR2 levels, both of which were suppressed by sc-BPNSs and DFO (Figure 6B and C). STAT3 knockdown reduced the expression of STAT3,* p*-STAT3 (Ser727), and TFR2. Importantly, sc-BPNSs further decreased TFR2 expression after STAT3 knockdown (Figure 6I and J). These findings suggest that although STAT3 participates in TFR2 regulation, sc-BPNSs can suppress TFR2 through mechanisms that are not fully dependent on STAT3.

Taken together, these results support the view that sc-BPNSs regulate iron homeostasis through both direct and indirect mechanisms. On the one hand, sc-BPNSs exert direct iron-chelating activity and reduce intracellular iron burden. On the other hand, sc-BPNSs modulate iron homeostasis-related pathways involving IRP1/TFR1 and STAT3/TFR2.

### sc-BPNSs attenuate inflammation and ferroptosis by disrupting the proinflammatory tubule-macrophage loop

In addition to inducing cell death, ferroptosis can also activate innate immune responses. To explore the immunomodulatory effects of sc-BPNSs, DEGs from the H/R + sc-BPNS group were intersected with the GOBP_ACUTE_INFLAMMATORY_RESPONSE gene set. This analysis revealed significant enrichment of inflammation-related genes regulated by sc-BPNSs (Figure S4A). Among these candidates, serum amyloid A (SAA) family members, including *Saa2* and *Saa4*, emerged as plausible mediators. Previous studies have shown that SAA can activate macrophage inflammatory programs, drive macrophages toward an M1-like phenotype, and induce NF-κB-related proinflammatory signaling 27.

**Figure 7:**
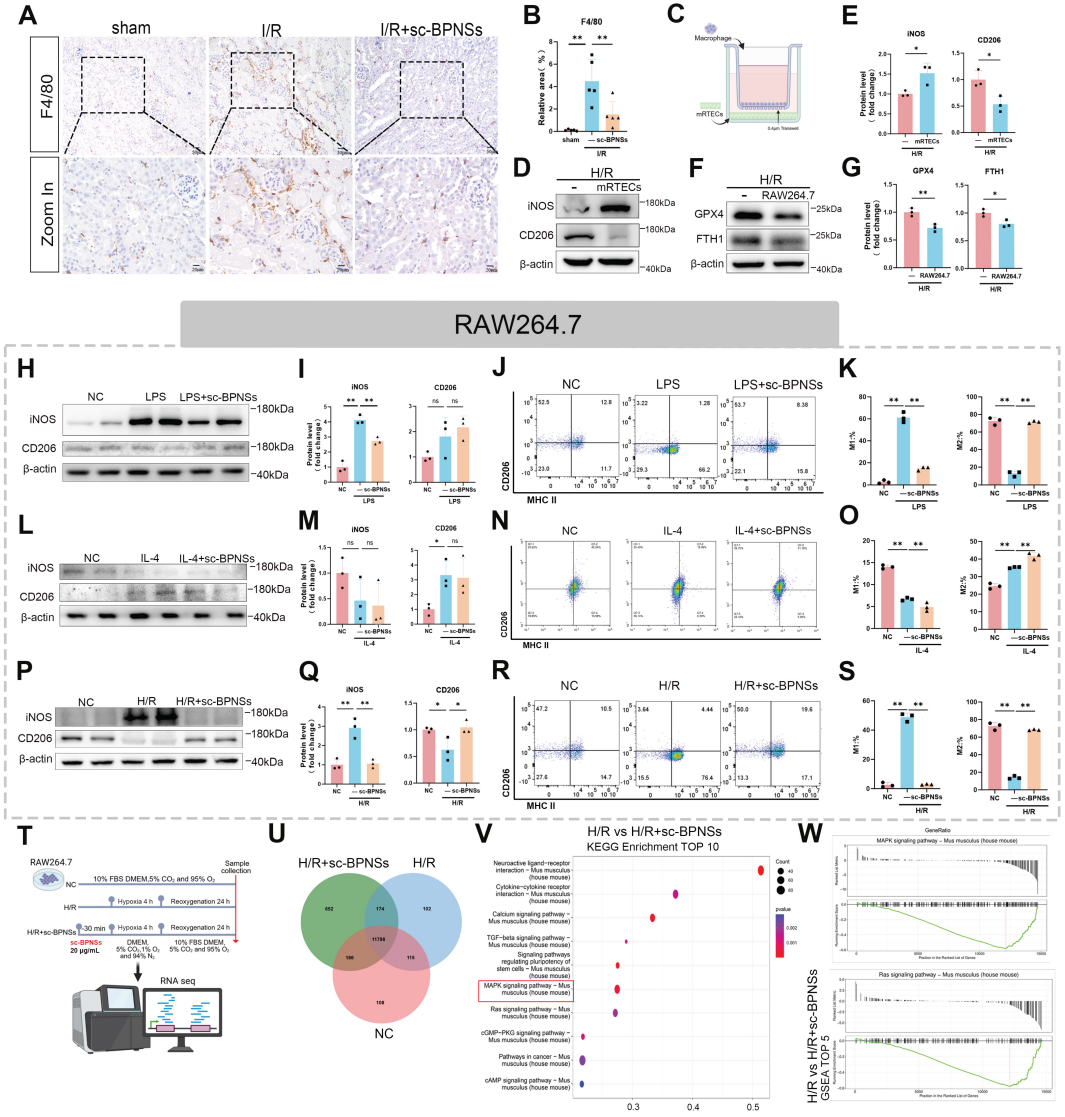
** sc-BPNSs attenuate inflammation and ferroptosis by disrupting the proinflammatory tubule-macrophage loop.** (A) Representative immunohistochemical staining images of F4/80 in renal tissues from each group. (B) Quantification of F4/80-positive macrophages (n = 5; **P < 0.01). (C) Schematic illustration of the mRTEC-RAW264.7 macrophage co-culture system. (D) Representative Western blot analysis of the M1 marker iNOS and the M2 marker CD206 in RAW264.7 cells under co-culture conditions. (E) Quantification of iNOS and CD206 protein levels under co-culture conditions (n = 3; *P < 0.05). (F) Representative Western blot analysis of GPX4 and FTH1 in mRTECs under co-culture conditions. (G) Quantification of GPX4 and FTH1 expression under co-culture conditions (n = 3; *P < 0.05, **P < 0.01). (H) Representative Western blot analysis of iNOS and CD206 in RAW264.7 cells stimulated with LPS (100 ng/mL) in the presence or absence of sc-BPNSs. (I) Quantification of iNOS and CD206 protein levels in the LPS-induced model (n = 3; **P < 0.01; ns, not significant). (J) Representative flow cytometry plots showing macrophage polarization under LPS stimulation. (K) Quantification of M1 and M2 fractions shown in panel J (n = 3; **P < 0.01). (L) Representative Western blot analysis of iNOS and CD206 in the IL-4 (20 ng/mL)-induced M2 polarization model in the presence or absence of sc-BPNS treatment. (M) Quantification of iNOS and CD206 expression in the IL-4 model (n = 3; ns, not significant). (N) Representative flow cytometry plots showing macrophage polarization under IL-4 stimulation. (O) Quantification of M1 and M2 fractions shown in panel N (n = 3; **P < 0.01). (P) Representative Western blot analysis of iNOS and CD206 in RAW264.7 cells subjected to H/R injury in the presence and absence of sc-BPNS treatment. (Q) Quantification of iNOS and CD206 protein levels in the H/R model (n = 3; *P < 0.05, **P < 0.01). (R) Representative flow cytometry plots showing macrophage polarization under H/R conditions. (S) Quantification of M1 and M2 fractions shown in panel R (n = 3; **P < 0.01). (T) Diagram of the RNA sequencing workflow. (U) Venn diagram showing 1,375 identified between DEGs between the H/R and H/R + sc-BPNSs groups. (V) KEGG pathway enrichment analysis indicating significant enrichment of MAPK signaling. (W) GSEA results demonstrating that sc-BPNS treatment significantly downregulates Ras and MAPK signaling pathways.

*In vivo*, immunohistochemical staining of I/R-injured kidneys showed substantial infiltration of F4/80⁺ macrophages. This infiltration was markedly reduced after sc-BPNS treatment (Figure 7A and B), indicating that sc-BPNSs attenuate macrophage accumulation in the injured kidney. To further investigate the interaction between tubular epithelial cells and macrophages under injury conditions, we used a Transwell co-culture system using H/R-exposed mRTECs and macrophages (Figure 7C). Compared with monoculture, co-culture shifted RAW264.7 cells toward an M1-like phenotype, as shown by increased nitric oxide synthase (iNOS) and decreased mannose receptor C-type 1 (CD206) expression (Figure 7D and E). Conversely, the presence of macrophages further reduced GPX4 and FTH1 levels in mRTECs (Figure 7F and G). These results suggest that macrophage-derived inflammatory signals aggravate ferroptosis-related injury in tubular epithelial cells, forming a reciprocal injury-inflammation loop.

The effects of sc-BPNSs on macrophage polarization were also tested under classical and alternative activation conditions. In the LPS-induced M1 polarization model, Western blotting showed that sc-BPNSs reduced iNOS expression, whereas CD206 expression changed little (Figure 7H and I). Flow cytometry further showed that sc-BPNSs decreased the M1-like fraction and increased the M2-like fraction (Figure 7J and K), consistent with a shift in macrophage polarization under LPS stimulation. In the IL-4-induced M2 polarization model, Western blotting showed no significant effect of sc-BPNSs on either iNOS or CD206 expression (Figure 7L and M). At the cellular population level, however, flow cytometry showed that sc-BPNSs decreased the M1-like fraction and further increased the M2-like fraction (Figure 7N and O). Thus, sc-BPNSs still altered macrophage subset distribution under IL-4 stimulation, although this change was not evident in bulk protein expression.

Because the AKI microenvironment is characterized by both hypoxic and inflammatory stress, macrophage polarization was further examined under H/R conditions. H/R markedly increased iNOS expression and reduced CD206 expression, whereas sc-BPNSs reversed these changes (Figure 7P and Q). Consistently, flow cytometric analysis showed that sc-BPNSs significantly decreased the M1 fraction and increased the M2 fraction under H/R conditions (Figure 7R and S), indicating that sc-BPNSs can simultaneously suppress proinflammatory polarization and promote a relatively reparative phenotype in an AKI-relevant stress setting. ROS probe staining confirmed that sc-BPNSs also reduced ROS levels in M1-polarized macrophages (Figure S4E).

To further elucidate the underlying mechanisms, RNA sequencing was performed in RAW264.7 cells treated with sc-BPNSs. The experimental design and correlation heatmap confirmed strong intragroup consistency (Figure 7T; Figure S4B). A total of 13,135 genes were commonly expressed across groups, with 2,070 DEGs identified among groups and 1,375 genes significantly altered between the H/R and H/R + sc-BPNSs groups (Figure 7U; Figure S4C). GO enrichment analysis of the H/R versus H/R + sc-BPNSs comparison revealed prominent enrichment in inflammatory signaling, extracellular matrix remodeling, ion channel activity, and apoptosis-related processes (Figure S4D). KEGG pathway analysis identified the MAPK signaling pathway as one of the most significantly enriched pathways following sc-BPNS intervention (Figure 7V). Gene set enrichment analysis (GSEA) further confirmed that sc-BPNSs suppressed Ras and MAPK pathway activity (Figure 7W).

Given the established role of Ras-MAPK signaling in macrophage activation and proinflammatory polarization 28, these transcriptomic data support the possibility that sc-BPNSs attenuate macrophage inflammatory responses through suppression of this pathway. This interpretation is consistent with the functional results showing reduced M1 polarization and inflammatory activation, and supports an inhibitory effect of sc-BPNSs on inflammatory amplification within the tubule-macrophage axis.

### sc-BPNSs modulate macrophage polarization through inhibition of the Ras-MAPK signaling pathway

**Figure 8:**
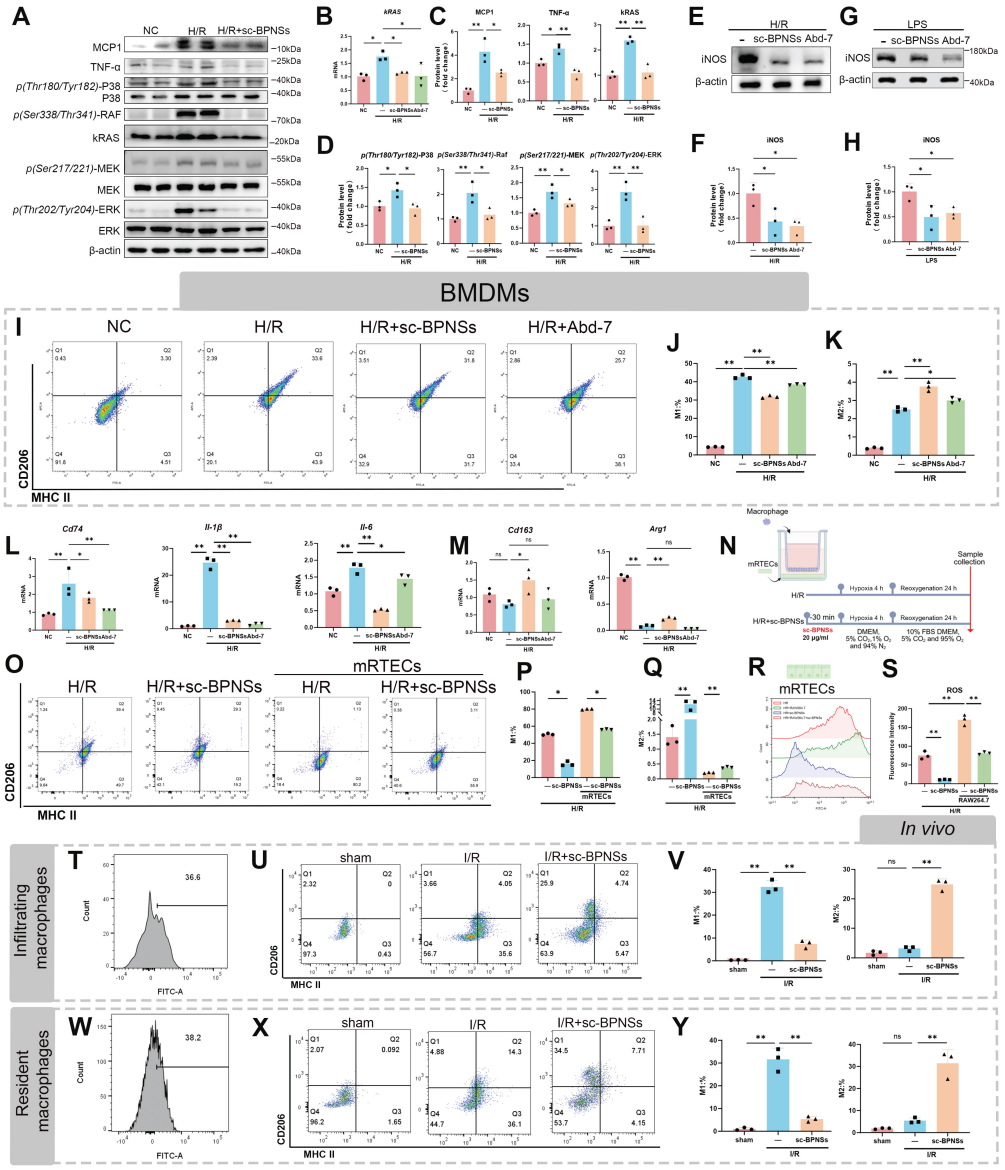
** sc-BPNSs modulate macrophage polarization through inhibition of the Ras-MAPK signaling pathway.** (A) Representative Western blot analysis of MCP-1, TNF-α, kRas, *p-*P38 (Thr180/Tyr182), total P38, *p-*RAF (Ser338/Thr341), *p-*MEK (Ser217/221), total MEK, *p-*ERK (Thr202/Tyr204), and total ERK in RAW264.7 macrophages under H/R conditions. (B) Quantitative PCR analysis of *kRas* mRNA expression (n = 3; *P < 0.05). (C) Quantification of MCP-1, TNF-α, and kRas protein levels in each group (n = 3; *P < 0.05, **P < 0.01). (D) Quantification of *p-*P38, *p-*RAF, *p-*MEK, and *p-*ERK protein expression (n = 3; *P < 0.05, **P < 0.01). (E) Representative Western blot analysis of iNOS in RAW264.7 cells treated with sc-BPNSs or the kRas inhibitor Abd-7 under H/R conditions. (F) Quantification of iNOS expression (n = 3; *P < 0.05). (G) Representative Western blot analysis of iNOS in LPS-stimulated macrophages treated with sc-BPNSs or Abd-7. (H) Quantification of iNOS levels under LPS stimulation (n = 3; *P < 0.05). (I) Representative flow cytometry plots of BMDMs under H/R conditions after treatment with sc-BPNSs or Abd-7. (J) Proportion of MHC II⁺ M1 macrophages (n = 3; **P < 0.01). (K) Proportion of CD206⁺ M2 macrophages (n = 3; **P < 0.01). (L) Quantitative analysis of the mRNA levels of the M1-associated markers *Cd86*, *Il1β*, and *Il6* in RAW264.7 cells under H/R conditions (n = 3; *P < 0.05, **P < 0.01). (M) Quantitative analysis of the mRNA levels of the M2-associated markers *Cd163* and *Arg1* in RAW264.7 cells under H/R conditions (n = 3; *P < 0.05, **P < 0.01). (N) Schematic illustration of the Transwell co-culture system, in which RAW264.7 cells were seeded in the upper insert and mRTECs were seeded in the lower well, followed by H/R treatment to simulate intercellular crosstalk. (O) Representative flow cytometry plots of macrophages cultured alone or co-cultured with mRTECs. (P, Q) Quantification of the M1 and M2 fractions shown in panel O (n = 3; *P < 0.05). (R) Representative ROS flow cytometry or fluorescence plots in mRTECs cultured alone or co-cultured with RAW264.7 cells under H/R conditions. (S) Quantification of ROS fluorescence intensity shown in panel R (n = 3; *P < 0.05). (T) Representative flow cytometric histogram showing uptake of FITC-sc-BPNSs in infiltrating macrophages, gated according to the strategy described in Figure 2J. (U) Representative flow cytometric dot plots showing the polarization profile of infiltrating macrophages in each group, analyzed by MHC II and CD206 expression. (V) Quantification of M1 and M2 populations among infiltrating macrophages (n = 3; **P < 0.01; ns, not significant). (W) Representative flow cytometric histogram showing uptake of FITC-sc-BPNSs in resident macrophages, gated according to the strategy described in Figure 2J. (X) Representative flow cytometric dot plots showing the polarization profile of resident macrophages in each group, analyzed by MHC II and CD206 expression. (Y) Quantification of M1 and M2 populations among resident macrophages (n = 3; **P < 0.01; ns, not significant).

To determine whether sc-BPNSs regulate macrophage polarization through inhibition of the Ras-MAPK pathway, inflammatory cytokines and Ras signaling proteins were first assessed in RAW264.7 cells under H/R conditions. H/R treatment significantly upregulated MCP-1, tumor necrosis factor-α (TNF-α) and Kirsten rat sarcoma viral oncogene homolog (kRas), along with downstream phosphorylated components, including *p-*P38 (Thr180/Tyr182), *p-*RAF (Ser338/Thr341), *p-*MEK (Ser217/221), and *p-*ERK (Thr202/Tyr204) (Figure 8A). sc-BPNS pretreatment reduced the abundance of kRas and the phosphorylation of downstream MAPK pathway proteins, indicating suppression of Ras-MAPK pathway activation. Quantitative analysis supported these protein-level changes (Figure 8C and D). At the mRNA level, qPCR showed that sc-BPNSs also lowered *kRas* expression (Figure 8B), suggesting that sc-BPNSs modulates *kRas* transcription and downstream pathway activation.

To examine whether Ras-MAPK signaling was involved in macrophage polarization, sc-BPNSs were compared with Abd-7, a specific kRas inhibitor. In the H/R model, both sc-BPNSs and Abd-7 decreased iNOS expression, while only sc-BPNSs increased CD206 levels (Figure 8E and F; Figure S4F and G). In the LPS-induced M1 polarization model, both treatments reduced iNOS expression, but neither significantly changed CD206 expression (Figure 8G and H; Figure S4H and I). This pattern suggests that Ras-MAPK inhibition contributes to the suppression of M1-like polarization, whereas sc-BPNSs may exert additional effects related to M2-associated features under specific injury conditions.

We further validated these observations in primary bone marrow-derived macrophages (BMDMs). Under H/R conditions, BMDMs showed an increased M1-like fraction and a decreased M2-like fraction. Both changes were partly reversed by sc-BPNSs and Abd-7, with sc-BPNSs showing a stronger reduction in the M1-like population (Figure 8I-K). Comparable trends were seen in primary macrophages stimulated with LPS or IL-4, although the extent of the response varied across treatments (Figure S4J-O). These data further support the involvement of Ras-MAPK signaling in sc-BPNS-mediated regulation of macrophage polarization, while also suggesting that additional mechanisms may be involved.

Consistent with these results, qPCR analysis in H/R-treated RAW264.7 cells showed that both sc-BPNSs and Abd-7 reduced the expression of the M1-associated genes *Cd86*,* Il1β*, and *Il6* (Figure 8L). Notably, only sc-BPNSs increased the M2-associated markers *Arg1* and *Cd163* (Figure 8M), indicating that sc-BPNSs reshape macrophage phenotype more broadly than simple Ras inhibition.

To evaluate the relevance of this effect in a tubule-macrophage interaction setting, a Transwell co-culture system was established using H/R-treated mRTECs and macrophages (Figure 8N). Compared with macrophages exposed to H/R alone, co-culture with injured mRTECs further increased the M1 fraction and reduced the M2 fraction, whereas sc-BPNS treatment partially reversed this shift (Figure 8O-Q). In parallel, co-culture with macrophages further aggravated H/R -induced ROS accumulation in mRTECs, while sc-BPNSs significantly reduced fluorescence intensity (Figure 8R and S). These results indicate that sc-BPNSs attenuate inflammatory amplification and oxidative crosstalk between tubular cells and macrophages.

Finally, using the kidney single-cell dissociation and gating strategy shown in Figure 2J, *in vivo* uptake and polarization status of renal macrophage subsets were examined after administration of FITC-sc-BPNSs. Flow cytometric analysis showed that both infiltrating and resident macrophages internalized sc-BPNSs *in vivo*, with FITC-positive fractions of approximately 36.6% and 38.2%, respectively (Figure 8T and W). In both macrophage populations, I/R markedly increased the M1 fraction and reduced the M2 fraction, whereas sc-BPNS treatment significantly decreased M1 polarization and promoted M2 polarization (Figure 8U, V, X, and Y). These findings are consistent with the *in vitro* results and further demonstrate that sc-BPNSs reprogram macrophage polarization in the AKI microenvironment *in vivo*.

Collectively, these data indicate that the effects of sc-BPNSs on macrophage polarization are partly mediated through inhibition of Ras-MAPK signaling but extend beyond simple Ras blockade and are closely associated with preferential tubular uptake and broader remodeling of the renal inflammatory microenvironment.

## Discussion

AKI remains a major clinical challenge, characterized by high morbidity and mortality, while lacking disease-directed treatment options. Disease progression is not driven by a single pathway; instead, oxidative stress, ferroptotic tubular injury, and dysregulated immune responses act in concert to amplify renal tubular epithelial cell damage and promote subsequent transition to CKD 29-31. This multilayered injury process may explain why current management remains largely supportive and why many single-target interventions have shown limited translational success. In this context, the present findings identify sc-BPNSs as a multifunctional nanoplatform capable of reducing iron availability, supressing ferroptosis, and attenuating inflammatory amplification in the injured kidney.

A central advance of this study is that the improved therapeutic performance of sc-BPNSs arises from restoration of the intrinsic surface chemistry of BPNSs rather than introduction of an entirely new material. Conventional BPNSs are chemically reactive but prone to spontaneous surface oxidation during preparation and processing, which compromises their antioxidant activity and metal coordination capacity 32, 33. By introducing a high-temperature hydrogen reduction step after conventional synthesis, surface oxides were removed, exposed phosphorus sites were restored, and material stability under biologically relevant conditions were improved.

Compared with conventional BPNSs, sc-BPNSs contain more exposed, unoxidized phosphorus atoms, enabling redox reactions with ROS at higher stoichiometric capacity and thereby achieving stronger ROS-scavenging efficiency (Figure 1). Removal of surface oxides may restore the reactivity of phosphorus atoms in BPNSs, thereby enhancing ROS clearance per unit mass and strengthening antioxidant capacity. Meanwhile, the exposed phosphorus atoms exhibited clear coordination ability with metal cations. This chelation capacity may be facilitated by restoration of phosphorus lone-pair reactivity after removal of surface oxygen, as supported by recent studies showing that metal-coordinated or protected BPNSs exhibit enhanced stability and increased metal ion coordination, including zinc- and magnesium-coordinated BPNSs 24, 34. Surface engineering may induce cleavage of P-O bonds on the surface of oxidized BPNSs and re-expose lone pairs of electrons on phosphorus atoms, thereby significantly enhancing coordination with metal ions such as Fe³⁺ and Fe²⁺. This surface chemical recovery is particularly relevant to AKI, where oxidative stress and redox-active iron are present in the same injured microenvironment.

Clear renal protection was observed after sc-BPNS treatment in the AKI models used in this study. In the I/R model, sc-BPNSs improved glomerular filtration, lowered sCr and BUN levels, and reduced tubular damage at both histological and molecular levels (Figure 2). Similar effects were seen in the Cis- and FA-induced AKI models. This suggests that sc-BPNSs are not limited to an ischemia-specific mechanism, but instead act on injury processes shared by different AKI etiologies, including oxidative stress, iron imbalance, and inflammatory amplification.

Another important finding is that sc-BPNSs showed injury-associated renal enrichment after systemic administration. Although signals were also detected in the liver, which is expected for intravenously delivered nanomaterials 35, renal accumulation was enhanced in AKI and was further supported by unilateral I/R experiments showing stronger signals in the injured kidney than in the contralateral kidney. This distribution pattern is consistent with the ultrathin two-dimensional (2D) structure and submicron lateral size of sc-BPNSs, as similar high-aspect-ratio nanosheets have been reported to access renal compartments after systemic administration. Prior studies have shown that 2D nanosheets, such as conventional BPNSs with a thickness of 3-4 nm and lateral size of approximately 400-600 nm, as well as graphene oxide with a lateral size of approximately 1 μm, can efficiently undergo glomerular filtration and accumulate in the renal cortex shortly after systemic administration 9, 36. One proposed mechanism suggests that such anisotropic nanostructures align with hydrodynamic flow and insert perpendicularly into glomerular basement membrane slits, thereby facilitating translocation 37. At the same time, AKI is known to alter renal hemodynamics, increase vascular permeability, and disrupt local barrier integrity, all of which may facilitate the entry and retention of circulating nanomaterials within injured renal tissue 38. Flow cytometric demonstrated that tubular epithelial cells constituted the major intrarenal population associated with sc-BPNS uptake, whereas infiltrating and resident macrophages showed detectable but lower FITC signals (Figures 2 and 8). These findings should not be interpreted as evidence of absolute strict kidney specificity. Rather, they suggest that sc-BPNSs can efficiently access the injured renal compartment, with tubular epithelial cells acting as the predominant material-associated target population *in vivo* (Figures 1 and 2).

At the mechanistic level, our data place ferroptosis inhibition at the center of sc-BPNS-mediated renal protection. Ferroptosis is characterized by iron-dependent lipid peroxidation and characteristic mitochondrial damage, and it has been implicated in AKI pathogenesis 16, 39. In the present study, sc-BPNSs ameliorated several ferroptosis-related abnormalities, including mitochondrial ultrastructural injury, loss of GPX4 and FTH1, and accumulation of lipid peroxidation products, such as 4-HNE, MDA, and lipid ROS. By contrast, sc-BPNSs showed only a limited effect on TUNEL positivity, suggesting that their renoprotective effect is more closely linked to suppression of iron-dependent lipid oxidative injury than to a broad anti-apoptotic effect. The NAC positive-control experiments and the *in vivo* 4-HNE staining results are also consistent with this ferroptosis-oriented interpretation (Figures 2 and 3).

A notable feature of sc-BPNSs is that they appear to act upstream of lipid peroxidation by limiting iron availability, rather than merely scavenging ROS after injury has occurred. In ferroptosis, Fe²⁺ is the more redox-active iron species because it directly participates in Fenton chemistry, generating hydroxyl radicals and promoting lipid peroxidation. Fe³⁺ is less directly reactive, but it contributes to transport- and storage-related iron pools and can be reduced to Fe²⁺ under oxidative or reductive conditions, thereby replenishing the labile iron pool 31. UV-Vis spectroscopy and XPS confirmed a direct interaction between sc-BPNSs and ferric ions, together with the formation of iron-associated complexes. The Fe 2p spectrum also showed partial conversion of Fe³⁺ to Fe²⁺, supporting a redox interaction during Fe³⁺ coordination. Consistent with these chemical data, FerroOrange staining and Prussian blue staining showed that sc-BPNSs decreased intracellular Fe²⁺ accumulation and renal iron deposition at the cellular and tissue levels. Functionally, sc-BPNSs protected tubular epithelial cells against both Fe²⁺- and Fe³⁺-induced injury (Figure 5). These findings suggest that sc-BPNSs reduce the bioavailability of redox-active iron, restore intracellular iron homeostasis, and thereby suppress iron-dependent lipid peroxidation. This interpretation is further supported by the TFR2 overexpression experiment, in which sc-BPNSs remained protective under enhanced iron influx, even when FTH1 was not fully restored (Figure 4). Thus, sc-BPNSs may partly compensate for impaired ferritin-dependent iron storage by lowering the bioavailable iron pool.

Although the intracellular fate of iron-associated sc-BPNSs was not directly traced in the present study, published evidence provides a plausible framework for their subsequent handling. BPNSs have been reported to enter cells through endocytic pathways and accumulate in lysosome-related compartments, whereas nanomaterials more generally may subsequently undergo intracellular trafficking and, in some cases, exocytotic clearance 40-42. Within this context, iron-associated sc-BPNSs may be processed through similar pathways after cellular uptake.

In parallel, sc-BPNSs were accompanied by coordinated remodeling of iron handling proteins, including reduced TFR1 and TFR2, restored FPN1 and FTH1, recovery of ACO1 activity, and attenuation of IRP1 binding to *Tfr1* mRNA. These changes indicate a shift toward reduced iron uptake and improved iron export and storage. Because oxidative stress can disrupt the [4Fe-4S] cluster of IRP1 and promote its RNA-binding apo form, the recovery of ACO1 activity and reduced IRP1-*Tfr1* mRNA binding suggest that sc-BPNSs may preserve IRP1 aconitase function by attenuating oxidative stress (Figure 6) 24, 34. By contrast, because TFR2 lacks a canonical IRE motif, its regulation is unlikely to be explained by the IRP1-IRE axis alone. The decline in *p*-STAT3 (Ser727) after sc-BPNS treatment, together with predicted STAT3-binding sites in the *Tfr2* promoter, supports a parallel STAT3-associated regulatory mechanism, consistent with STAT3 acting as a pro-injury regulator of TFR2 expression. Thus, sc-BPNSs may suppress TFR2 expression, at least in part, by inhibiting oxidative stress-associated STAT3 phosphorylation (Figure 6) 16, 39, 43.

Taken together, these findings suggest that sc-BPNSs restore iron homeostasis through coordinated mechanisms rather than a simple linear pathway. Direct iron buffering reduces the bioavailability of labile iron, whereas antioxidative activity contributes to IRP1- and STAT3-associated remodeling of iron handling proteins. These two processes likely act in concert to limit iron influx, promote iron storage and export, and suppress ferroptotic lipid peroxidation.

Beyond ferroptosis control, sc-BPNSs reprogrammed immune responses. Injured tubules can release DAMPs, cytokines, chemokines, and extracellular vesicle-associated signals that promote macrophage recruitment and pro-inflammatory polarization, whereas activated macrophages further aggravate tubular oxidative stress, lipid peroxidation, and ferroptotic injury. This reciprocal tubule-macrophage amplification loop has been reported in AKI and is considered a major mechanism that drives injury propagation and delays renal repair 44-46. Consistently, the present co-culture data showed that H/R-injured tubular cells promoted a proinflammatory shift in macrophages, while macrophages in turn aggravated the loss of GPX4 and FTH1 in tubular cells (Figure 7). Previous studies have also shown that injured tubular epithelial cells can communicate with macrophages through extracellular vesicles, thereby promoting M1 macrophage activation in kidney injury 46, 47.

Transcriptomic analysis further identified SAA family members as plausible mediators of this crosstalk, consistent with previous studies showing that SAA can drive macrophage inflammatory activation and M1-like polarization 27. However, because direct blocking experiments were not performed, SAA should be regarded as a candidate mediator rather than a proven causal factor.

sc-BPNSs weakened this tubule-macrophage injury loop at multiple levels. In RAW264.7 cells and primary BMDMs, sc-BPNSs suppressed M1 polarization and promoted M2-associated features under H/R-, LPS-, or IL-4-related stress conditions. Transcriptomic analysis, GSEA, and validation experiments further indicated that Ras-MAPK signaling is one mechanistically relevant pathway involved in this effect (Figures 7 and 8). This is consistent with previous studies showing that MAPK pathways, including ERK, JNK, and p38, participate in macrophage inflammatory activation and M1 polarization 48. *In vivo* flow cytometry further showed that both infiltrating and resident macrophages internalized sc-BPNSs in injured kidneys and underwent polarization changes consistent with the *in vitro* findings. Taken together, the macrophage data support that sc-BPNSs regulate macrophage polarization, at least in part, by suppressing Ras-MAPK signaling. Other mechanisms are also likely involved, including reduced inflammatory signaling from injured tubular epithelial cells and attenuation of oxidative and iron-driven inflammatory stress.

From the materials standpoint, these findings show that removing surface oxygen species changes the way BPNSs behave in biological systems. Conventional BPNSs are prone to surface oxidation, which can reduce their chemical reductivity and weaken metal coordination. By contrast, sc-BPNSs preserve more exposed, electron-rich phosphorus sites, thereby improving their ability to scavenge ROS and buffer iron.

For future translational development, sc-BPNSs have several favorable features: efficient access to injured kidneys, pronounced uptake by tubular epithelial cells, combined inhibition of ferroptosis and inflammatory activation, and a surface chemistry that may be suitable for further functional modification. Nevertheless, several limitations should be acknowledged. First, although the present data support iron buffering as a key mechanism, renal iron distribution, iron speciation, and the intracellular fate of iron-associated sc-BPNSs were not directly traced *in vivo*. Second, the current design does not fully separate direct iron buffering from antioxidative remodeling of IRP1- and STAT3-associated pathways, partly due to the lack of a well-matched non-chelating BPNS control. Third, the precise soluble mediators linking tubular injury to macrophage reprogramming remain to be validated. Finally, sc-BPNSs were administered before injury induction in the current study, therefore, their efficacy as a post-injury therapeutic intervention remains to be determined. Future studies should incorporate spatially resolved and speciation-resolved iron analysis, intracellular tracing of iron-associated sc-BPNSs, more rigorous control materials, validation of candidate mediators such as SAA, and long term safety and pharmacokinetic evaluation in translationally relevant models.

## Conclusion

Collectively, this study demonstrates that sc-BPNSs protect against AKI through coupled suppression of ferroptotic injury and inflammatory immune responses. Surface cleaning restores the chemical reactivity of BPNSs and enhances their antioxidant and iron-chelating capacities. As a result, sc-BPNSs reduce redox-active iron burden, attenuate tubular ferroptotic injury, and limit proinflammatory macrophage activation. These findings identify sc-BPNSs as a surface chemistry-optimized nanoplatform with promising therapeutic potential for AKI.

## Supplementary Material

Supplementary figures.

## Figures and Tables

**Table 1:** Primer sequences used for qPCR

Gene	Forward (5' to 3')	Reverse (5' to 3')
mus *Tfr2*	TCAGCGTGCTACACCTCAAAGC	CTCAATGAGGCTGACGAGAAGG
mus *Tfr1*	GAAGTCCAGTGTGGGAACAGGT	CAACCACTCAGTGGCACCAACA
mus *Fth1*	GCCGAGAAACTGATGAAGCTGC	GCACACTCCATTGCATTCAGCC
mus *Fpn1*	CCATAGTCTCTGTCAGCCTGCT	CTTGCAGCAACTGTGTCACCGT
mus *Cd74*	GCTGGATGAAGCAGTGGCTCTT	GATGTGGCTGACTTCTTCCTGG
mus *Mmd*	CACGATGGGATTTTCTCCAGCC	CAAACAGGTGCCAGATGGCATG
mus *kRas*	TTTGCTGGCAGTGATTGGAGGAC	ACCACCTGTGAAGATTACACGGAAG
mus *Cd86*	CCGTGGAGACGCAAGCTTAT	CAACTTTTGCTGGTCCTGCC
mus *Il1β*	GTGAAATGCCACCTTTTGACAGTG	CCAGGTCAAAGGTTTGGAAGC
mus *Il6*	GGGACTGATGCTGGTGACAA	ACAGGTCTGTTGGGAGTGGT
mus *Cd163*	GATCCATGACAAGTGGGGCA	GCTGGAGTTAGCCCATCCAA
mus *Arg1*	ACATTGGCTTGCGAGACGTA	ATCACCTTGCCAATCCCCAG

**Table 2:** siRNA sequences

siRNA	Design Sequence
*Stat3*-siRNA target sequence	AAAUGAAGGUGGUGGAGAATT
	UUCUCCACCACCUUCAUUUTT
*Irp1*-siRNA target sequence	GGUAAUAGCAUAUGCGAUUTT
	AAUCGCAUAUGCUAUUACCTT
*Control*-siRNA target sequence	UUCUCCGAACGUGUCACGUTT
	ACGUGACACGUUCGGAGAATT

## Data Availability

The data supporting the findings of this study are available from the corresponding author upon reasonable request. Source data underlying the main figures and supplementary materials are provided in the Supporting information.

## Abbreviations

ABTS: 2,2’-azino-bis(3-ethylbenzothiazoline-6-sulfonic acid)
ACO1: aconitase 1
AFM: atomic force microscopy
AKI: acute kidney injury
ALT: alanine aminotransferase
APC: allophycocyanin
Arg1: arginase 1
AST: aspartate aminotransferase
BCA: bicinchoninic acid
BMDMs: bone marrow-derived macrophages
BPNSs: black phosphorus nanosheets
BUN: blood urea nitrogen
CCK-8: Cell Counting Kit-8
CCR2: C-C motif chemokine receptor 2
CD11b: cluster of differentiation 11b
CD45: cluster of differentiation 45
CD74: cluster of differentiation 74
CD86: cluster of differentiation 86
CD163: cluster of differentiation 163
CD206: cluster of differentiation 206
Cis: cisplatin
CK-MB: creatine kinase-MB
CKD: chronic kidney disease
Cy5.5: cyanine 5.5
DAB: 3,3’-diaminobenzidine
DAMPs: damage-associated molecular patterns
DAPI: 4’,6-diamidino-2-phenylindole
DCF: dichlorofluorescein
DCFH-DA: 2’,7’-dichlorodihydrofluorescein diacetate
DEGs: differentially expressed genes
DFO: deferoxamine
DLS: dynamic light scattering
DMEM: Dulbecco’s modified Eagle medium
DPPH: 2,2-diphenyl-1-picrylhydrazyl
ECL: enhanced chemiluminescence
EDS: energy-dispersive X-ray spectroscopy
EpCAM: epithelial cell adhesion molecule
ERK: extracellular signal-regulated kinase
FA: folic acid
F4/80: macrophage surface marker F4/80
FBS: fetal bovine serum
Fe^2+^: ferrous ion
Fe^3+^: ferric ion
FITC: fluorescein isothiocyanate
FPN1: ferroportin 1
FTH1: ferritin heavy chain 1
GBM: glomerular basement membrane
GFR: glomerular filtration rate
GO: Gene Ontology
GPX4: glutathione peroxidase 4
GSH: reduced glutathione
GSSG: oxidized glutathione
H&E: hematoxylin and eosin
H/R: hypoxia/reoxygenation
HRP: horseradish peroxidase
HRTEM: high-resolution transmission electron microscopy
ICG: indocyanine green
IHC: immunohistochemistry
IL-1β: interleukin-1β
IL-4: interleukin-4
IL-6: interleukin-6
iNOS: inducible nitric oxide synthase
IRE: iron-responsive element
IRP1: iron regulatory protein 1
I/R: ischemia/reperfusion
IRI: ischemia/reperfusion injury
KEGG: Kyoto Encyclopedia of Genes and Genomes
KIM-1: kidney injury molecule-1
kRas: Kirsten rat sarcoma viral oncogene homolog
LPS: lipopolysaccharide
M-CSF: macrophage colony-stimulating factor
MAPK: mitogen-activated protein kinase
MCP-1: monocyte chemoattractant protein-1
MDA: malondialdehyde
MEK: mitogen-activated protein kinase kinase
MHC II: major histocompatibility complex class II
MMD: monocyte to macrophage differentiation-associated
mRTECs: mouse renal tubular epithelial cells
NAC: N-acetyl-L-cysteine
NC: normal control
Ngal: neutrophil gelatinase-associated lipocalin
NMP: N-methyl-2-pyrrolidone
PAS: periodic acid-Schiff
PBS: phosphate-buffered saline
PE: phycoerythrin
PEG: polyethylene glycol
PerCP: peridinin-chlorophyll-protein complex
PVDF: polyvinylidene fluoride
qPCR: quantitative polymerase chain reaction
RAW264.7: mouse macrophage cell line RAW264.7
RIP: RNA immunoprecipitation
RNA-seq: RNA sequencing
ROS: reactive oxygen species
sCr: serum creatinine
sc-BPNSs: surface-cleaned black phosphorus nanosheets
SD: standard deviation
SDS-PAGE: sodium dodecyl sulfate-polyacrylamide gel electrophoresis
siRNA: small interfering RNA
STAT3: signal transducer and activator of transcription 3
TBST: Tris-buffered saline with Tween-20
TECs: tubular epithelial cells
TEM: transmission electron microscopy
TFR1: transferrin receptor 1
TFR2: transferrin receptor 2
tGSH: total glutathione
TSS: transcription start site
TUNEL: terminal deoxynucleotidyl transferase dUTP nick-end labeling
UV-Vis: ultraviolet-visible spectroscopy
XPS: X-ray photoelectron spectroscopy
XRD: X-ray diffraction
