# Supplementary material for: Surface-cleaned black phosphorus nanosheets chelate iron and rebalance the ferroptosis-immune axis in acute kidney injury
Source: Theranostics. 2026 Jul 20;16(14):8151–79. doi: 10.7150/thno.129807 (PMC13440469; doi:10.7150/thno.129807)
Supplement: Supplementary file 1 [file thnov16p8151s1.pdf]

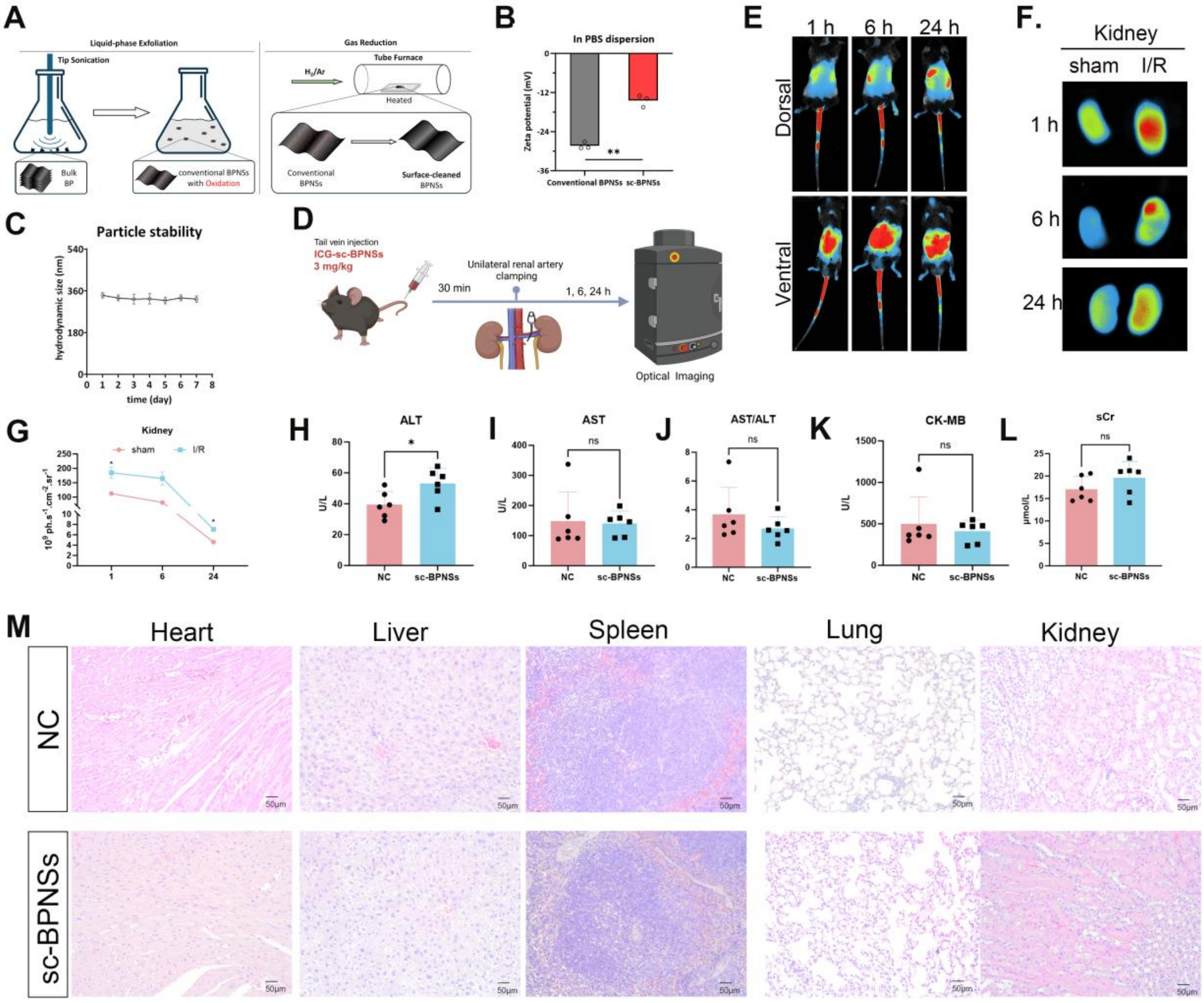

Supplementary Figure 1 | Complementary *in vivo* biodistribution analysis in a unilateral left renal I/R model and biosafety evaluation of long term sc-BPNSs administration *in vivo*.

(A) Schematic diagram of sc-BPNSs preparation process.

(B) Zeta potential of conventional BPNSs and sc-BPNSs measured in PBS dispersion.

(C) Hydrodynamic size of sc-BPNSs in PBS over 7 days, showing good colloidal stability.

(D) Schematic illustration of the unilateral left renal I/R model and the in vivo optical imaging workflow. Mice were intravenously injected with ICG-sc-BPNSs (3 mg/kg) via the tail vein, subjected to 30 min of unilateral left renal artery clamping, and then imaged at 1, 6, and 24 h.

(E) Representative whole body fluorescence images acquired at 1, 6, and 24 h after intravenous injection of ICG-sc-BPNSs in mice subjected to unilateral I/R, shown from the dorsal and ventral views.

(F) Representative ex vivo fluorescence images of kidneys collected at 1, 6, and 24 h after intravenous injection of Cy5.5-sc-BPNSs in the unilateral I/R model.

(G) Quantification of renal fluorescence intensity shown in (F) (n = 3; *$P < 0.05$).

(H) ALT levels were slightly elevated in the sc-BPNSs group compared with the control group (n = 6, *$P < 0.05$), but remained within the physiological range.

(I) AST levels showed no significant difference between groups (n = 6; ns, no significance).

(J) AST/ALT ratio exhibited no significant change following sc-BPNSs treatment (n = 6; ns, no significance).

(K) CK-MB levels showed no significant difference between the sc-BPNSs treated and control groups (n = 6, ns, no significance).

(L) sCr levels were comparable between groups (n = 6; ns, no significance).

(M) Representative H&E staining images of major organs (heart, liver, spleen, lung, and kidney) from mice after 28 days of sc-BPNSs administration (3 mg/kg, i.v., every 3 days). No evident histopathological abnormalities were observed.

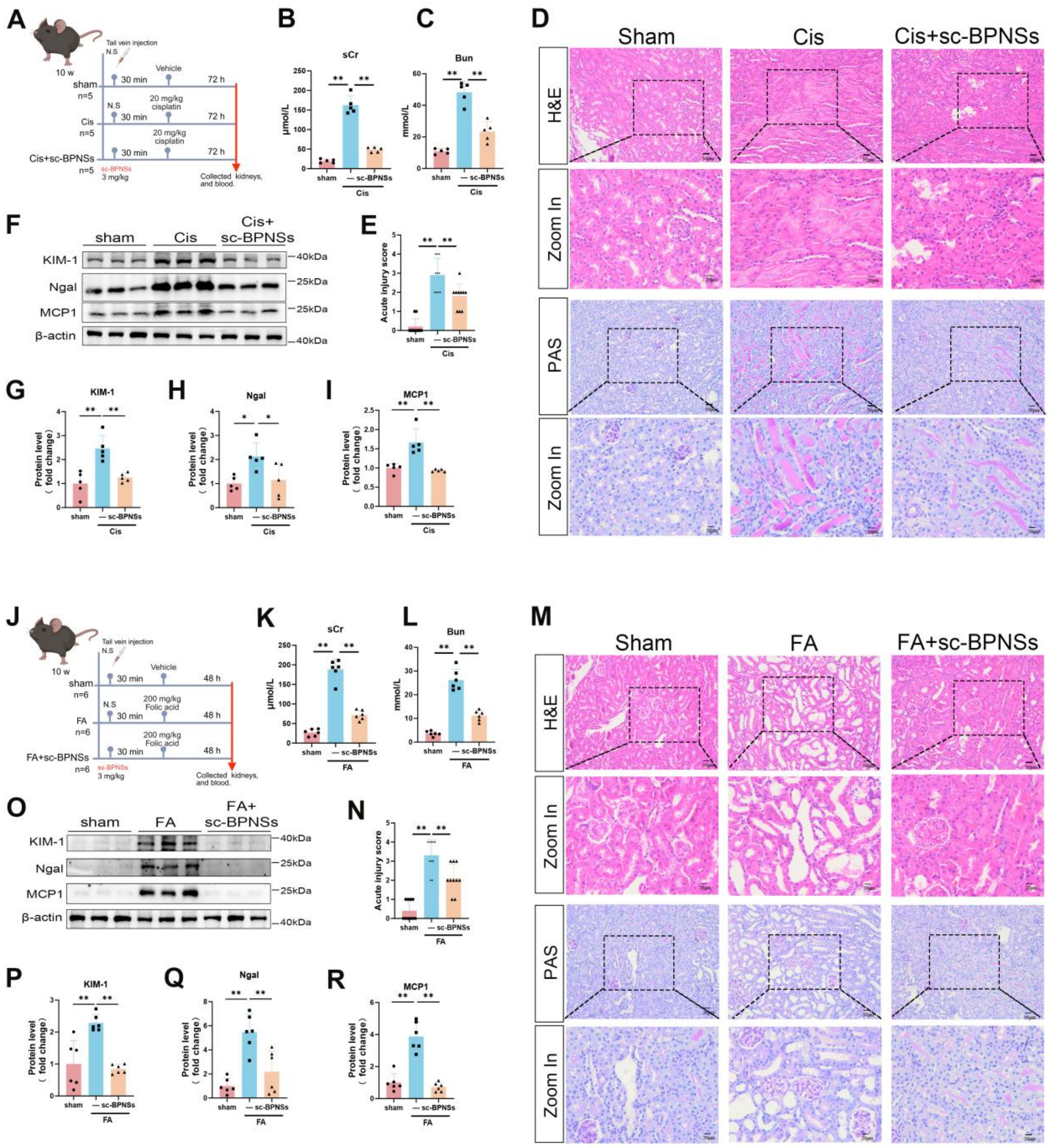

Supplementary Figure 2 | sc-BPNSs exert similar renoprotective effects in Cisplatin and Folic acid induced AKI models.

(A) Schematic illustration of the Cis induced AKI model and treatment protocol. Mice were intravenously injected with sc-BPNSs (3 mg/kg), followed 30 min later by intraperitoneal injection of Cis (20 mg/kg), and blood and kidney tissues were collected 72 h later.

(B, C) sCr and BUN levels in each group (n = 5; $**P < 0.01$).

(D) Representative H&E and PAS staining images of kidney sections from each group, with enlarged views of the boxed regions.

(E) Acute injury scores in each group (n = 10 fields per group; $**P < 0.01$).

(F) Western blot analysis of KIM-1, Ngal, and MCP1 protein expression in kidney tissues from

each group.
(G–I) Densitometric quantification of KIM-1, Ngal, and MCP1 protein expression (n = 5; $^{**}P < 0.01$, $^{*}P < 0.05$).
(J) Schematic illustration of the FA induced AKI model and treatment protocol. Mice were intravenously injected with sc-BPNSs (3 mg/kg), followed 30 min later by intraperitoneal injection of FA (200 mg/kg), and blood and kidney tissues were collected 48 h later.
(K, L) sCr and BUN levels in each group (n = 6; $^{**}P < 0.01$).
(M) Representative H&E and PAS staining images of kidney sections from each group, with enlarged views of the boxed regions.
(N) Acute injury scores in each group (n = 10 fields per group; $^{**}P < 0.01$).
(O) Western blot analysis of KIM-1, Ngal, and MCP1 protein expression in kidney tissues from each group.
(P–R) Densitometric quantification of KIM-1, Ngal, and MCP1 protein expression (n = 6; $^{**}P < 0.01$).

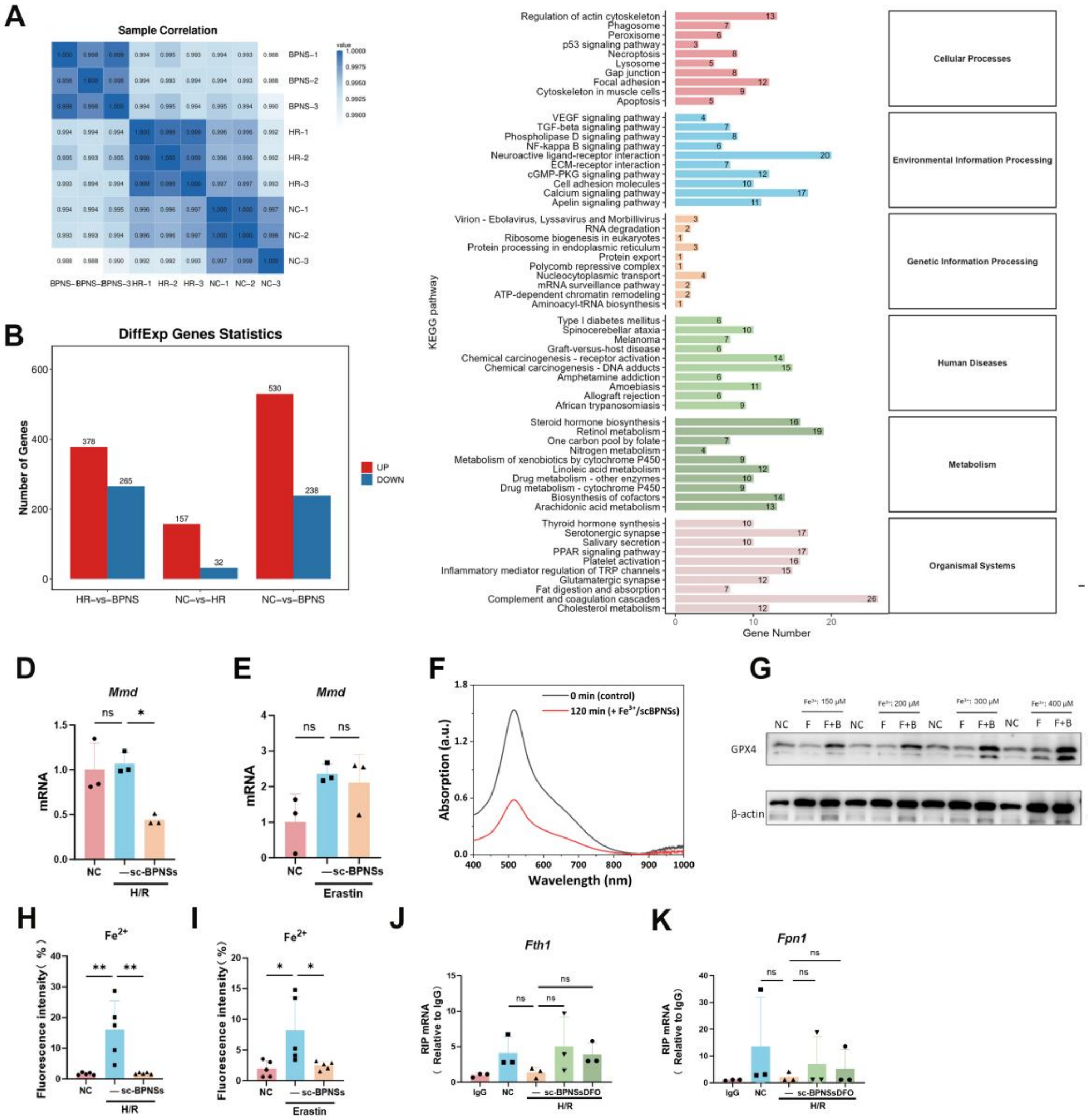

Supplementary Figure 3 | Transcriptomic and functional validation of the ferroptosis-inhibitory mechanism of sc-BPNSs.
(A) Heatmap of sample correlations from RNA seq showing high intra-group consistency among

NC, H/R, and H/R+sc-BPNSs groups (n = 3 per group).
(B) Bar graph illustrating the number of DEGs between H/R and H/R+sc-BPNSs groups; sc-BPNSs treatment led to 643 DEGs, including 378 upregulated and 265 downregulated genes.
(C) KEGG pathway enrichment analysis of DEGs between H/R and H/R+sc-BPNSs groups revealed significant enrichment in oxidative stress related pathways, including arachidonic acid metabolism and peroxisome signaling.
(D) qRT-PCR analysis of *Mmd* mRNA expression under H/R conditions showed a moderate but significant reduction upon sc-BPNSs treatment (n = 3, *$P < 0.05$; ns, no significance).
(E) qRT-PCR analysis of *Mmd* expression under Erastin treatment showed no significant change with sc-BPNSs (n = 3; ns, no significance).
(F) DPPH radical-scavenging assay of Fe^{3+}-bound sc-BPNSs (Fe^{3+}/sc-BPNSs), showing the absorption spectra of the 0 min control and the 120 min Fe^{3+}/sc-BPNSs reaction system.
(G) Western blot analysis of GPX4 protein expression in mRTECs exposed to varying concentrations of Fe^{3+} (150, 200, 300, 400 μM), with or without sc-BPNSs; sc-BPNSs preserved GPX4 levels under iron overload conditions, indicating ferroptosis inhibition.
(H) FerroOrange fluorescence staining showing reduced intracellular Fe^{2+} levels in H/R-exposed mRTECs treated with sc-BPNSs (n = 3, **$P < 0.01$).
(I) FerroOrange staining in Erastin induced ferroptotic cells also demonstrated decreased Fe^{2+} accumulation following sc-BPNSs treatment (n = 3, *$P < 0.05$).
(J) RIP analysis of the binding between IRP1 and *Fth1* mRNA. RIP was performed using an anti-IRP1 antibody, with IgG as a negative control, and the enrichment of *Fth1* mRNA in the immunoprecipitated complexes was quantified by qPCR. Data are presented as *Fth1* mRNA levels relative to IgG (n = 3; ns, no significance).
(K) RIP analysis of the binding between IRP1 and *Fpn1* mRNA. Data are presented as *Fpn1* mRNA levels relative to IgG (n = 3; ns, no significance).

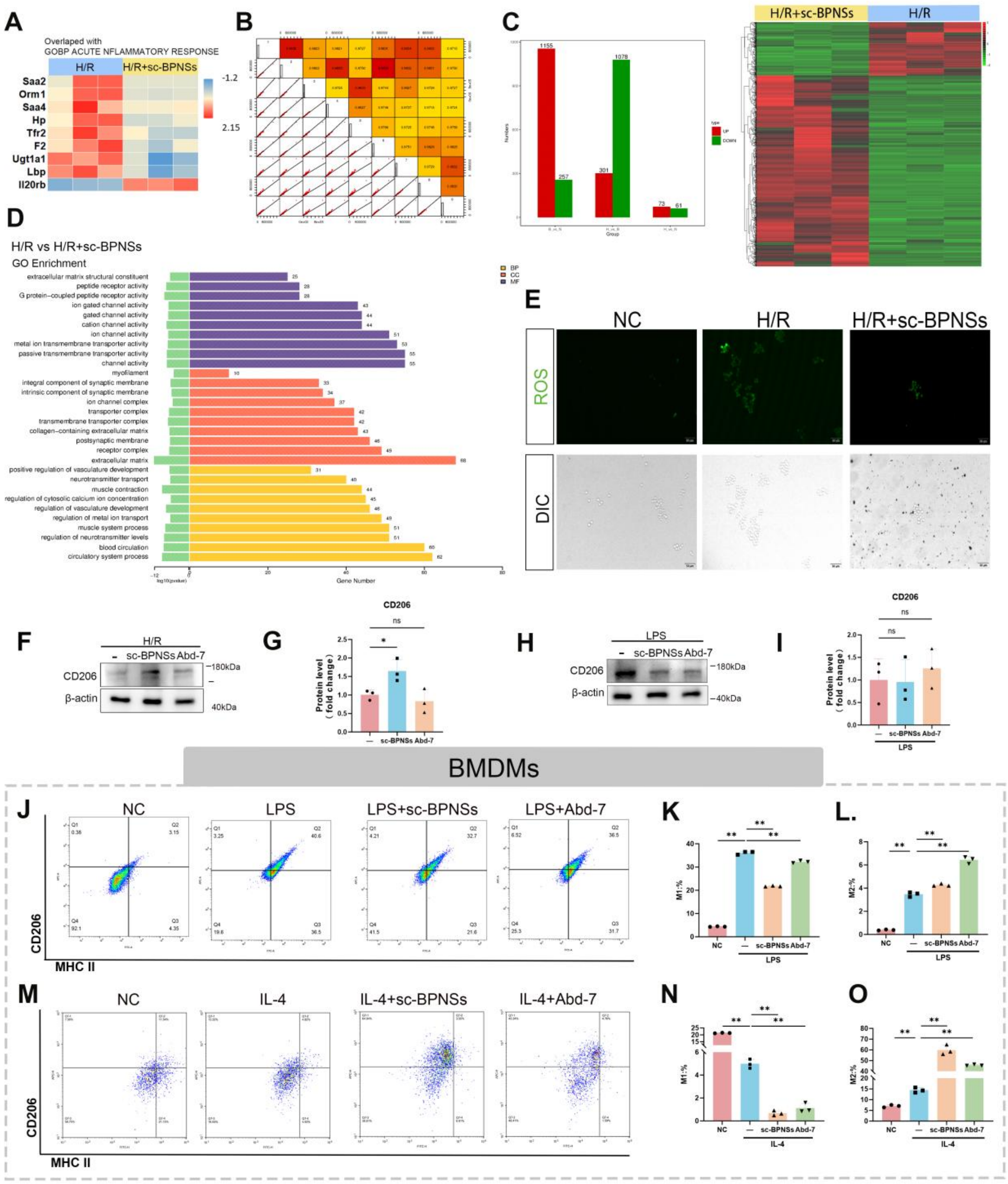

Supplementary Figure 4 | Transcriptomic analysis reveals that sc-BPNSs regulate macrophage polarization by attenuating inflammatory responses.

(A) Heatmap of differentially expressed genes (DEGs) overlapping with the Gene Ontology Biological Process term "acute inflammatory response" (GOBP_ACUTE_INFLAMMATORY_RESPONSE). Inflammatory mediators were significantly upregulated under H/R conditions and markedly suppressed following sc-BPNSs treatment.

(B) Pearson correlation heatmap of gene expression profiles among NC, H/R, and H/R+sc-BPNSs groups, demonstrating high intra-group and inter-group reproducibility.

(C) Bar graph summarizing the number of DEGs across group comparisons: the largest number was found in sc-BPNSs vs. NC (B_vs_N), followed by H/R vs. sc-BPNSs (H_vs_B), with H/R vs. NC (H_vs_N) showing the fewest changes. (Right) Hierarchical clustering heatmap of DEGs between H/R and H/R+sc-BPNSs groups; red indicates upregulation and green indicates

downregulation. sc-BPNSs reversed the transcriptional alterations induced by H/R.

(D) GO enrichment analysis of DEGs between H/R and H/R+sc-BPNSs groups showed significant enrichment in biological processes such as inflammatory signaling, extracellular matrix remodeling, membrane channel regulation, and apoptotic pathways.

(E) Intracellular ROS levels in RAW264.7 macrophages under H/R conditions were reduced following sc-BPNSs treatment, as measured by ROS specific fluorescent probes.

(F–G) Western blot analysis and quantification of CD206 expression in RAW264.7 cells under H/R conditions after treatment with sc-BPNSs or Abd-7 (n = 3, *$P < 0.05$; ns, not significant).

(H–I) Western blot analysis and quantification of CD206 expression in RAW264.7 cells under LPS stimulation after treatment with sc-BPNSs or Abd-7 (n = 3; ns, not significant).

(J–L) Representative flow cytometry plots and quantification of the M1 and M2 fractions in BMDMs under LPS stimulation after treatment with sc-BPNSs or Abd-7 (n = 3, **$P < 0.01$).

(M–O) Representative flow cytometry plots and quantification of the M1 and M2 fractions in BMDMs under IL-4 stimulation after treatment with sc-BPNSs or Abd-7 (n = 3, **$P < 0.01$).

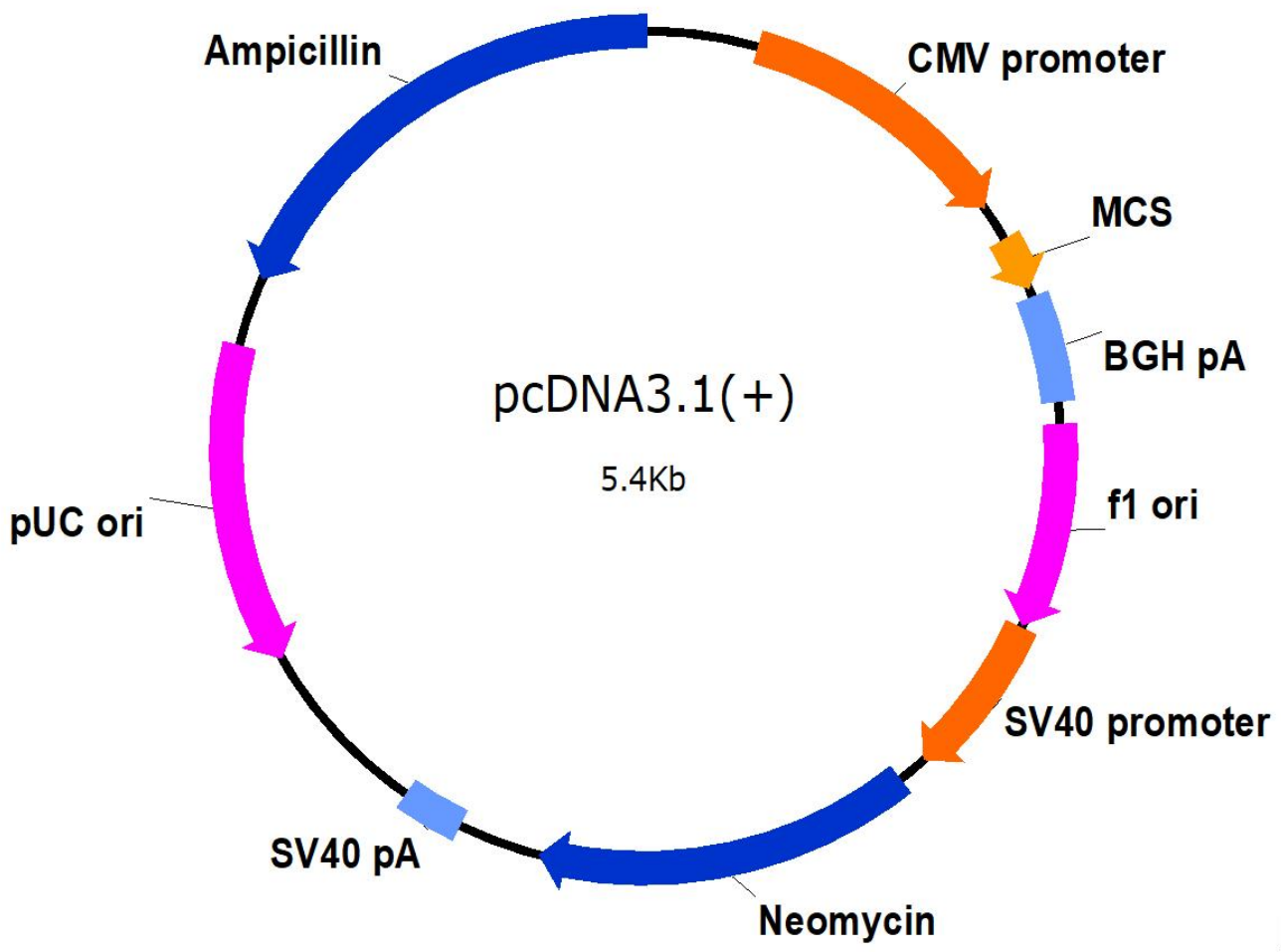

Supplementary Figure 5 | Schematic map of the pcDNA3.1(+) vector used for construction of the *Tfr2* overexpression plasmid.
